# RIGI, TLR7, and TLR3 Genes Were Predicted to Have Immune Response Against Avian Influenza in Indigenous Ducks

**DOI:** 10.3389/fmolb.2021.633283

**Published:** 2021-12-14

**Authors:** Aruna Pal, Abantika Pal, Pradyumna Baviskar

**Affiliations:** ^1^ West Bengal University of Animal and Fishery Sciences, Kolkata, India; ^2^ Indian Institute of Technology Kharagpur, Kharagpur, India; ^3^ St. Jude Children’s Research Hospital, Memphis, TN, United States

**Keywords:** *Anas platyrhynchos*, avian influenza, RIGI, TLR3, TLR7

## Abstract

Avian influenza is a disease with every possibility to evolve as a human-to-human pandemic arising out of frequent mutations and genetic reassortment or recombination of avian influenza (AI) virus. The greatest concern is that till date, no satisfactory medicine or vaccines are available, leading to massive culling of poultry birds, causing huge economic loss and ban on export of chicken products, which emphasizes the need to develop an alternative strategy for control of AI. In the current study, we attempt to explore the molecular mechanism of innate immune potential of ducks against avian influenza. In the present study, we have characterized immune response molecules such as duck TLR3, TLR7, and RIGI that are predicted to have potent antiviral activities against the identified strain of avian influenza through *in silico* studies (molecular docking) followed by experimental validation with differential mRNA expression analysis. Future exploitation may include immunomodulation with the recombinant protein, and transgenic or gene-edited chicken resistant to bird flu.

## Introduction

Ducks are reported to be relatively resistant to common poultry diseases, including viral disease, compared to chicken (Pal et al., 2017), and are commonly asymptomatic to avian influenza virus infection (Kim et al., 2009; Fleming-Canepa et al., 2019; CDC, Centre for Disease control and Prevention, 2021). There is clear lack of further systematic characterization of the indigenous ducks at the molecular level. In an effort to understand, we have studied this as a first step. Hence, there is an urgent need to explore the innate immune response genes, particularly against viral infection.

Avian influenza is caused by single-stranded RNA virus, which is negatively stranded, and belongs to Orthomyxoviridae family (WHO, 2019). It is commonly known as bird flu since birds are the main host. Based on the antigenic differences, two surface proteins, namely, hemagglutinin and neuraminidase of avian influenza virus have been mostly subtyped and the nomenclature is provided accordingly. Till date, 18 subtypes of HA (H1–H18) and 11NA (N1–N11) have been detected (Tong et al., 2013; Ying et al., 2014). H5, H7, and H9 were observed to be the most pathogenic subtypes of bird. Most of the H5 and H7 subtypes were regarded as highly pathogenic avian influenza (HPAI) virus, owing to the higher incidence and mortality of birds. The greatest concern is the lack of definite treatment or vaccination due to frequent mutation and reassortment of viral strain, regarded as antigenic shift and antigenic drift (Webster and Govorkova, 2014; WHO, 2020). Due to massive culling of birds in the affected area and the ban on the export of poultry products, the WHO has regarded avian influenza as one of the most economically effected zoonotic diseases (WHO, 2018b). It has been reported that in West Bengal during the 2008 outbreak, there was loss of 500.42 crores, 6 percent of the poultry population was culled, and five lakh families were affected. In Manipur at the 2007 outbreak, the loss due to the disease has been found to be 14 per cent of the total value of livestock outputs in the entire state. More than three lakh birds were culled, and 24 tonnes of poultry feed was destroyed post-flu (Otte et al., 2008a). Vietnam estimated the direct losses of 109 village and backyard producers with flock sizes smaller than 50 birds at US$69 (VND1084000) per farm (Otte et al., 2008a). An average loss of US$22 was estimated per household from the loss of birds in Egypt (Otte et al., 2008a).

The basic mechanism of host immunity against viral infection is generally different from that of other infectious agents such as bacteria and protozoa. Viruses utilize the host immune mechanism for their infection and further survival, thus allowing it to act as hijackers. Accordingly, viruses employ the host cellular machinery for living normal cells through the process of invasion; multiplication within the host might in turn kill, damage, or change the cells, and make the individual sick (Maarouf et al., 2018). The molecular mechanism of replication of avian influenza virus involves certain proteins. The HA protein present from the surface of the AI virus aids in recognition and binding to sialic acid on the surface of host cells, thereby aiding in the entry of the virus in the host cell (Matrosovich et al., 2009a). Following the binding, virus particles are endocytosed, leading to endosome maturation, and pH is lowered, resulting in the conformational change in HA, thereby causing fusion of the endosome and virion membranes. The viral M2 protein (matrix 2) acts as an ion channel for further lowering of the pH of the viral particle. This leads to the dissociation of the M1 protein (matrix 1) virion “shell” in such a way that the eight vRNPs [NP (nucleoprotein)-coated and polymerase complex (PB1, PA, and PB2)-bound viral RNAs] are released into the cytosol (Basler and Aguilar, 2008a). In the next step, the viral RNPs transport into the cell nucleus wherein accessory cellular components necessary for influenza viral replication and transcription are present. Following the process of genome replication, transcription, and protein synthesis, NEP (nuclear export protein) and M1 act to traffic newly synthesized vRNPs out of the nucleus, into the cytoplasm, and to the plasma membrane, leading to the assembly of progeny virions. At this stage, several viral proteins contribute to budding, including M1 and M2. In the next step, NA aids in the removal of sialic acid from glycoproteins in both the viral and cell membranes, resulting in the prevention of the interaction between HA and host cell receptors, and release of new infectious virus particles (Medina and García-Sastre, 2011). NS1 (nonstructural protein 1) plays a role within the infected cell in order to counteract innate host–cell defense systems, for example, interferon (IFN), which may otherwise limit efficient virus replication (Hale et al., 2008).

As the virus gets an entry in the body, an immune response is triggered, followed by local inflammatory signaling. Innate immune reaction is initially activated by conserved pathogen-associated molecular pattern (PAMP), pattern recognition receptors (PRRs), retinoic acid–inducible gene (RIG)-I like receptors, MDA5, LGP 2, and toll-like receptor (TLRs) such as TLR3 and TLR7 (Kannaki et al., 2010). Viral nucleic acid binds to these receptors expressed on macrophages, microglia, dendritic cells, and astrocytes; releases type-I interferon (IFN-I); and helps in the production of interferon-stimulated genes (ISGs) (Ali et al., 2019). Interferon-I upregulates antiviral proteins, and accordingly, peripheral immune cells are stimulated and alter endothelial tight junction (Otte et al., 2008b). It has been observed that the absence of IFN_I signaling leads to the prevention of microglial differentiation and decrease of peripheral myeloid cell patrolling (Otte et al., 2008b).

TLR7 is a member of the Toll-like receptor family, which recognizes single-stranded RNA in endosomes, which is a common feature of viral genomes (Manglani and McGavern, 2018). TLR7 can recognize GU-rich single-stranded RNA. TRL7 was reported to have influences on viral infection in poultry and has been regarded as a vital component of antiviral immunity, particularly in ducks (Manglani and McGavern, 2018). RIG-I (retinoic acid–inducible gene I) or RIG-I–like receptor dsRNA helicase enzyme is part of the RIG-I–like receptor family, which also includes MDA5 and LGP2. These have been reported to function as a pattern recognition receptor that is a sensor for viruses such as influenza A, others such as Sendai virus, and Flavivirus (Matrosovich et al., 2009b). RIG-I typically recognizes short 5′ triphosphate uncapped double-stranded or single-stranded RNA (Basler and Aguilar, 2008b). RIG-I and MDA5 are the viral receptors, acting through a common adapter MAVS and trigger an antiviral response through type-I interferon response. RIG1 is an important gene conferring antiviral immunity for ducks, particularly avian influenza (Matrosovich et al., 2009b).

TLR3 is another member of the toll-like receptor (TLR) family. Infectious agents express PAMP (pathogen-associated molecular patterns), which is readily recognized by TLR3, which in turn secretes cytokines responsible for effective immunity. It recognizes dsRNA associated with a viral infection, and induces the activation of IRF3, unlike all other toll-like receptors which activate NF-κB (Kannakiet al., 2010). IRF3 ultimately induces the production of type I interferons, which is ultimately responsible for host defense against viruses (Kell and Gale, 2015). In our lab, earlier we had studied immunogenetics against bacterial disease with identified immune response molecule such as CD14 gene in goat (Vercammen et al., 2008a; Schlee et al., 2009), cattle (Stetson and Medzhitov, 2006), and buffalo (Pal and Chatterjee, 2009; Pal et al., 2013). We reported for the first time the role of mitochondrial cytochrome B gene for immunity in sheep (Pal et al., 2011) and immune-response genes in Haringhata Black chicken (Pal et al., 2014).

Indigenous duck population in the Indian subcontinent was observed to have better immunity against viral infections, and so far, no systematic studies were undertaken. Certain reports revealed that ducks were mostly asymptomatic and were better resistant to avian influenza infection. Thus, the present study was conducted with the aim of molecular characterization of immune response genes (TLR3, TLR7, and RIGI) of duck, providing the initial proteomics study and prediction of the binding site with multiple strains of avian influenza virus through *in silico* studies (molecular docking), establishment of disease-resistant genes of ducks through quantitative PCR, and experimental validation of the identified genes through differential mRNA expression profiling of the identified gene with respect to healthy and challenged embryonated eggs as *in vitro* studies.

## Materials and Methods

### Animals, Sample Collection, and RNA Isolation

#### Birds

Duck samples were collected from different agro-climatic regions of West Bengal, India, from farmer’s herd. The chicken breeds such as Haringhata Black and Aseel were maintained in the university farm (West Bengal University of Animal and Fishery Sciences). Samples from other poultry species such as guineafowl and goose were also collected from the university farm. Samples from turkey and quail were collected from State Poultry farm, Animal Resource Development Dept, Tollygunge, Govt. of West Bengal, India. The birds were vaccinated against routine diseases such as Ranikhet disease and fowl pox. Six male birds (aged 4–5 months) were considered under each group for this study and are maintained under uniform managemental conditions.

All experiments were conducted in accordance with relevant guidelines and regulations of the Institutional Animal Ethics Committee, and all experimental protocols were approved by the Institutional Biosafety Committee, West Bengal University of Animal and Fishery Sciences, Kolkata.

The total RNA was isolated from the ileocecal junction of duck, Haringhata Black chicken, Aseel, and other poultry species such as guineafowl and goose, using RiboPure Kit (Invitrogen), following the manufacturer’s instructions and was further used for cDNA synthesis (Schlee et al., 2009; Pal et al., 2011).

#### Materials

Taq DNA polymerase, 10X buffer, and dNTP were purchased from Invitrogen, and SYBR Green qPCR Master Mix (2X) was obtained from Thermo Fisher Scientific Inc. (PA, United States). L-Glutamine (Glutamax 100x) was purchased from Invitrogen corp., (Carlsbad, CA, United States). Penicillin-G and streptomycin were obtained from Amresco (Solon, OH, United States). Filters (Millex GV. 0.22 µm) were purchased from Millipore Pvt. Ltd., (Billerica, MA, United States). All other reagents were of analytical grade.

### Synthesis, Confirmation of cDNA, and PCR Amplification of TLR3, RIGI, and TLR7 Genes

The 20 μl reaction mixture contained 5 μg of total RNA, 0.5 μg of oligo dT primer (16–18 mer), 40 U of ribonuclease inhibitor, 10 M of dNTP mix, 10 mM of DTT, and 5 U of MuMLV reverse transcriptase in the reverse transcriptase buffer. The reaction mixture was gently mixed and incubated at 37°C for 1 h. The reaction was stopped by heating the mixture at 70°C for 10 min and chilled on ice. The integrity of the cDNA was checked by PCR. To amplify the full-length open reading frame (ORF) of the gene sequence, a specific primer pair was designed based on the mRNA sequences of *Gallus gallus* by DNASTAR software. The primers have been listed in Table 1. 25 μl of the reaction mixture contained 80–100 ng cDNA, 3.0 μl 10X PCR assay buffer, 0.5 μl of 10 mM dNTP, 1 U Taq DNA polymerase, 60 ng of each primer, and 2 mM MgCl2. PCRs were carried out in a thermocycler (PTC-200, MJ Research, United States) with the following cycling conditions: initial denaturation at 94°C for 3 min, denaturation at 94°C for 30 sec, and varying annealing temperature (as mentioned in Table 1) for 35 sec, and extension at 72°C for 3 min was carried out for 35 cycles followed by final extension at 72°C for 10 min.

**TABLE 1:** List of primers used for amplification of TLR3, RIG1, and TLR7 genes in indigenous duck.

Gene	Primer	Product length	Annealing temp
Primers used for amplification of TLR3 for duck			
Duck TLR3.1	FP: TGG​AAA​ACA​ATG​TCA​AAT​CAG	450	49.9
	RP: TCACGGAGGTTCTTCAG		
Duck TLR3.2	FP: TCCGTGAGCTTGTGTTGT	460	50.2
	RP: AGATGTTTGAGCCTGGAC		
Duck TLR3.3	FP: GATAAATTCGCTCACTGG	436	48.8
	RP: TCTAAGGCTTGGAACGA		
Duck TLR3.4	FP: TCA​GCA​ATA​ACA​ACA​TAG​CAA​ACA	456	51.8
	RP: GGG​TCG​CAT​TAA​GCC​AAC​T		
Duck TLR3.5	FP: ATA​TAC​CTG​GAT​TGC​AGT​CTC​AGT	650	52.7
	RP: CTG​GGC​TGG​CCA​CTT​CAA​G		
Primers used for amplification of RIG1 for duck			
Duck RIG1.1	FP: CTG​CAG​TGC​TAC​CGC​CGC​TAC​ATC	460	59.6
	RP: TAT​CCG​ACC​GAC​AGA​GAC​ATT​CAA		
Duck RIG1.2	FP:AAAGATGTTGACAGTGAAATG	402	50.8
	RP: TCC​TTG​AAC​AGA​GTA​TCC​TT		
Duck RIG1.3	FP: CAG​GAC​GAA​AGG​CGA​AAG​TT	448	53.8
	RP: TGT​ATG​TCA​AGG​TAG​GAG​CAG​AGA		
Duck RIG1.4	FP: ATC​CCT​TTG​CAG​CCA​TTA​TCC	585	55.2
	RP: CGCGCCCCATCAAAACAC		
Duck RIG1.5	FP: TAA​CTA​CAT​AAA​GCC​AGG​TG	448	50.4
	RP: TAC​TTT​AGG​TTT​TAT​TTC​TTT​C		
Duck RIG1.6	FP: CCA​GAA​GGA​AAG​AAA​TAA​AAC​C	416	52.3
	RP: TGG​TGG​GTA​CAA​GTT​GGA​CAT		
Primers used for amplification of TLR7 of duck			
Duck TLR7.1	FP: TCA​AGC​ATA​TTC​ATG​AAG​ACT​TT	513	58.4
	RP: TGGGCCCCAACCTGACAG		
Duck TLR7.2	FP: TTGAGAATGGCAGTTTTG	500	48.8
	RP: AGC​CTT​TGA​ATG​TAT​CTT​A		
Duck TLR7.3	FP: ACA​TTC​AAA​GGC​TTT​TTA​TTC​CT	754	52.4
	RP: TAT​TGC​ATT​ACC​TGA​CAA​GTT​GAG		
Duck TLR7.4	FP: GAT​GCC​TCA​ACT​TGT​CAG​GTA​ATG	751	53.5
	RP: TTT​TCG​GGG​AAG​CTA​GAT​TTC​TT		
Duck TLR7.5	FP: CTA​GCT​TCC​CCG​AAA​ATG​TCA​T	736	54.8
	RP: TTC​TGC​ACA​GCC​TTT​TCC​TCA​G		
Duck TLR7.6	FP: AGC​GCC​TTC​TAG​ATG​AAA​A	400	48.8
	RP: TTT​TAG​TTT​ATG​AGA​TTT​TAT​TAT		
List of primers used for QPCR study			
β-Actin	FP: 5′-GAG​AAA​TTG​TGC​GTG​ACA​TCA-3′	152	60
	RP: 5′-CCT​GAA​CCT​CTC​ATT​GCC​A-3′		
TLR2	FP: 5′CAT​TCA​CCA​TGA​GGC​AGG​GAT​AG-3′	157	60
	RP: 5′-GGT​GCA​GAT​CAA​GGA​CAC​TAG​GA-3′		
TLR4	FP: 5′-TTC​AGA​ACG​GAC​TCT​TGA​GTG​G-3′	131	60
	RP: 5′-CAA​CCG​AAT​AGT​GGT​GAC​GTT​G-3′		
TLR7	5′-TTG​CTG​CTG​TTG​TCT​TGA​GTG​AG-3′	182	60
	5′-AAC​AAC​AGT​GCA​TTT​GAC​GTC​CT-3′		
*Bu-1*	5′-GGC​TGT​TGT​GTC​CTC​ACT​CAT​CT-3′	106	60
	5′-CAC​CAC​CGA​CAT​TGT​TAT​TCC​AT-3′		

*The final and complete sequence is obtained by joining the fragments of the amplified products of the gene consecutively.

1TLR2, Toll-like receptor 2; TLR4, Toll-like receptor 4; TLR7, Toll-like receptor 7; Bu-1, chicken B-cell marker chB6.

### cDNA Cloning and Sequencing

PCR amplicons verified by 1% agarose gel electrophoresis were purified from gel using Gel Extraction Kit (Qiagen GmbH, Hilden, Germany) and ligated into a pGEM-T easy cloning vector (Promega, Madison, WI, United States) following the manufacturer’s instructions. The 10 μl of the ligated product was directly added to 200 μl competent cells, heat shock was given at 42°C for 45 s in a water bath, and cells were then immediately transferred on chilled ice for 5 min, and SOC was added. The bacterial culture was pelleted and plated on the LB agar plate containing ampicillin (100 mg/ml) added to the agar plate @ 1: 1000, IPTG (200 mg/ml) and X-Gal (20 mg/ml) for blue-white screening. Plasmid isolation from overnight-grown culture was done by the small-scale alkaline lysis method. Recombinant plasmids were characterized by PCR using gene-specific primers and restriction enzyme digestion based on the reported nucleotide sequence for cattle. The enzyme EcoR I (MBI Fermentas, United States) is used for fragment release. Gene fragment insert in the recombinant plasmid was sequenced by an automated sequencer (ABI prism) using the dideoxy chain termination method with T7 and SP6 primers (Chromous Biotech, Bangalore).

### Sequence Analysis

The nucleotide sequence so obtained was analyzed for protein translation, sequence alignments, and contig comparisons by DNASTAR version 4.0, Inc., United States. The novel sequence was submitted to the NCBI GenBank, and the accession number was obtained, which is available in a public domain now.

### Study of Predicted TLR3, TLR7, and RIG1 Peptides Using Bioinformatic Tools

The predicted peptide sequence of TLR3, TLR7, and RIG1 of indigenous duck was derived by Edit sequence (Lasergene Software, DNASTAR) and then aligned with the peptide of other chicken breed and avian species using Megalign sequence Programme of Lasergene Software (DNASTAR). Prediction of the signal peptide of the CD14 gene was conducted using the software (Signal P 3.0 Sewer-prediction results, Technical University of Denmark). Estimation of leucine percentage was conducted manually from the predicted peptide sequence. Di-sulfide bonds were predicted using suitable software (http://bioinformatics.bc.edu/clotelab/DiANNA/) and by homology search with other species.

The protein sequence-level analysis study was carried out with specific software (http://www.expasy.org./tools/blast/) for the determination of leucine-rich repeats (LRRs), leucine zipper, N-linked glycosylation sites, detection of leucine-rich nuclear export signals (NESs), and detection of the position of the GPI anchor. The detection of leucine-rich nuclear export signals (NESs) was carried out with NetNES 1.1 Server, Technical University of Denmark. The analysis of O-linked glycosylation sites was carried out using NetOGlyc 3.1 server (http://www.expassy.org/), whereas the N-linked glycosylation site was detected by NetNGlyc 1.0 software (http://www.expassy.org/). The detection of leucine-zipper was conducted through Expassy software, Technical University of Denmark (Pal et al., 2020). Regions for alpha-helix and beta-sheet were predicted using NetSurfP-Protein Surface Accessibility and Secondary Structure Predictions, Technical University of Denmark (Schlee et al., 2009; Pal et al., 2018a). Domain linker prediction was done according to the software developed (Pal et al., 2019a). The LPS-binding site (Glick, 1977) and LPS-signaling sites (Petersen et al., 2009) were predicted based on homology studies with other polypeptide species.

### Three-Dimensional Structure Prediction and Model Quality Assessment

The templates which possessed the highest sequence identity with our target template were identified by using PSI-BLAST (http://blast.ncbi.nlm.nih.gov/Blast). The homology modeling was used to build a 3D structure based on homologous template structures using PHYRE2 server (Ebina et al., 2009). The 3D structures were visualized by PyMOL (http://www.pymol.org/), which is an open-source molecular visualization tool. Subsequently, the mutant model was generated using the PyMoL tool. Swiss PDB Viewer was employed for controlling energy minimization. The structural evaluation along with a stereochemical quality assessment of predicted model was carried out by using the SAVES (Structural Analysis and Verification Server), which is an integrated server (http://nihserver.mbi.ucla.edu/SAVES/). The ProSA (Protein Structure Analysis) webserver (https://prosa.services.came.sbg.ac.at/prosa) was used for refinement and validation of the protein structure (Cunningham et al., 2000). The ProSA was used for checking model structural quality with potential errors, and the program shows a plot of its residue energies and Z-scores which determine the overall quality of the model. The solvent accessibility surface area of the IR genes was generated by using NetSurfP server (http://www.cbs.dtu.dk/services/NetSurfP/) (Muroi et al., 2002). It calculates relative surface accessibility, Z-fit score, the probability for Alpha-Helix, probability for beta-strand and coil score, *etc*. TM align software was used for the alignment of 3D structure of IR protein for different species and RMSD estimation to assess the structural differentiation (Kelley et al., 2015). The I-mutant analysis was conducted for mutations detected to assess the thermodynamic stability (Wiederstein and Sippl, 2007). PROVEAN analysis was conducted to assess the deleterious nature of the mutant amino acid (Pal et al., 2018b).

### Molecular Docking

Molecular docking is a bioinformatic tool used for *in silico* analysis for the prediction of the binding mode of a ligand with a protein 3D structure. PatchDock is an algorithm for molecular docking based on the shape complementarity principle (Zhang and Skolnick, 2005). The PatchDock algorithm was used to predict ligand–protein docking for surface antigen for avian influenza (H antigen and NA antigen) with the molecules for innate immunity against viral infections such as TLR3, TLR7, and RIG1. FireDock was employed for further confirmation (Capriotti et al., 2005). The amino acid sequence for the surface antigen (hemagglutinin and neuraminidase) from different strains of avian influenza was retrieved from gene bank. Hemagglutinin segment 4 sequence was collected from the Indian subcontinent as H5N1 (Acc no. KR021385, Protein id. AKD00332), H4N6 (Acc no. JX310059, Protein id. AF082958), H6N2 (Acc no. KU598235, Protein id. AMH93683), and H9N2 (Acc no. 218091, Protein id. AAG53040). Neuraminidase segment 6 was collected from the Indian subcontinent as H5N1 (Acc no. KT867346, Protein id. ALK80150), H4N6 (Acc no. JX310060, Protein id. AF082959), and H6N2 (Acc no. KU598237, Protein id. AMH93685) to be employed as the ligand. The receptor molecules employed were TLR3 (Gene bank accession number KX865107, NCBI, and derived protein as ASW23003), RIGI (Gene bank accession number KX865107, protein ASW23002 from NCBI), and TLR7 (Gene bank Accession no. MK986726, NCBI) for duck sequenced and characterized in our lab.

### Assessment of Antigenic Variability Among Different Strains of Avian Influenza

MAFFT software (Choi and Chan, 2015) was employed for the detection of amino acid variability and construction of phylogenetic tree for different strains of avian influenza detected in duck in the Indian subcontinent.

The amino acid sequence for the surface antigen (hemagglutinin and neuraminidase) from different strains of avian influenza was retrieved from gene bank. Hemagglutinin segment 4 sequence was collected from the Indian subcontinent as H5N1 (Acc no. KR021385, Protein id. AKD00332), H4N6 (Acc no. JX310059, Protein id. AF082958), H6N2 (Acc no. KU598235, Protein id. AMH93683), and H9N2 (Acc no. 218091, Protein id. AAG53040).

Neuraminidase segment 6 was collected from the Indian subcontinent as H5N1 (Acc no. KT867346, Protein id. ALK80150), H4N6 (Acc no. JX310060, Protein id. AF082959), and H6N2 (Acc no. KU598237, Protein id. AMH93685.1).

### Protein–Protein Interaction Network Depiction

In order to understand the network of TLR3, TLR7, and RIG1 peptides, we performed analysis submitting FASTA sequences to STRING 9.1 (Schneidman-Duhovny et al., 2005). Confidence scoring was used for functional analysis. Interactions with score <0.3 are considered as low confidence, scores ranging from 0.3 to 0.7 are classified as medium confidence, and scores >0.7 yield high confidence. The functional partners were depicted.

KEGG analysis also depicts the functional association of TLR3, TLR7, and RIG1 peptides with other related proteins (KEGG: Kyoto Encyclopedia of Genes and Genomes–GenomeNet, https://www.genome.jp/kegg/).

### Real-Time PCR

An equal amount of RNA (quantified by Qubit fluorometer, Invitrogen), wherever applicable, were used for cDNA preparation (Superscript III cDNA synthesis kit; Invitrogen). All qRT-PCR reactions were conducted on the ABI 7500 fast system. Each reaction consisted of 2 µl cDNA template, 5 µl of 2X SYBR Green PCR Master Mix, 0.25 µl each of forward and reverse primers (10 pmol/μl), and nuclease-free water for a final volume of 10 µl. Each sample was run in duplicate. Analysis of real-time PCR (qRT-PCR) was performed by the delta-delta-Ct (^ΔΔ^Ct) method. The primers used for QPCR analysis have been listed as per Table 1.

Studies were also conducted for differential mRNA expression profiling of TLR3, TLR7, and RIGI as an *in vitro* study in embryonic fibroblast cell of both chicken (Haringhata Black breed) and duck (Bengal duck) after the challenge study with the H5N1 strain of avian influenza virus in comparison to control in BSL3 lab. Samples were collected after 72 h of infection and subjected to RNA isolation, and the same steps were followed as explained earlier.

### Comparison of TLR3, TLR7, and RIG1 Structures of Indigenous Ducks With Respect to Chicken

Nucleotide variation for the proteins was detected from their nucleotide sequencing, and amino acid variations were estimated (DNASTAR). The 3D structure of the derived protein was estimated for both indigenous ducks and chicken by Pymol software. The PDB structure of the respective proteins was derived from *PHYRE* software (Mashiach et al., 2008). We also employed *Modeller software* for protein structural modeling (Katoh and Standley, 2013) for better confirmation. Alignment of the structure of TLR3, TLR7, and RIGI duck with chicken was conducted by TM Align software (Szklarczyk et al., 2015).

## Results

### Molecular Characterization of TLR3 Gene

**FIGURE 1:**
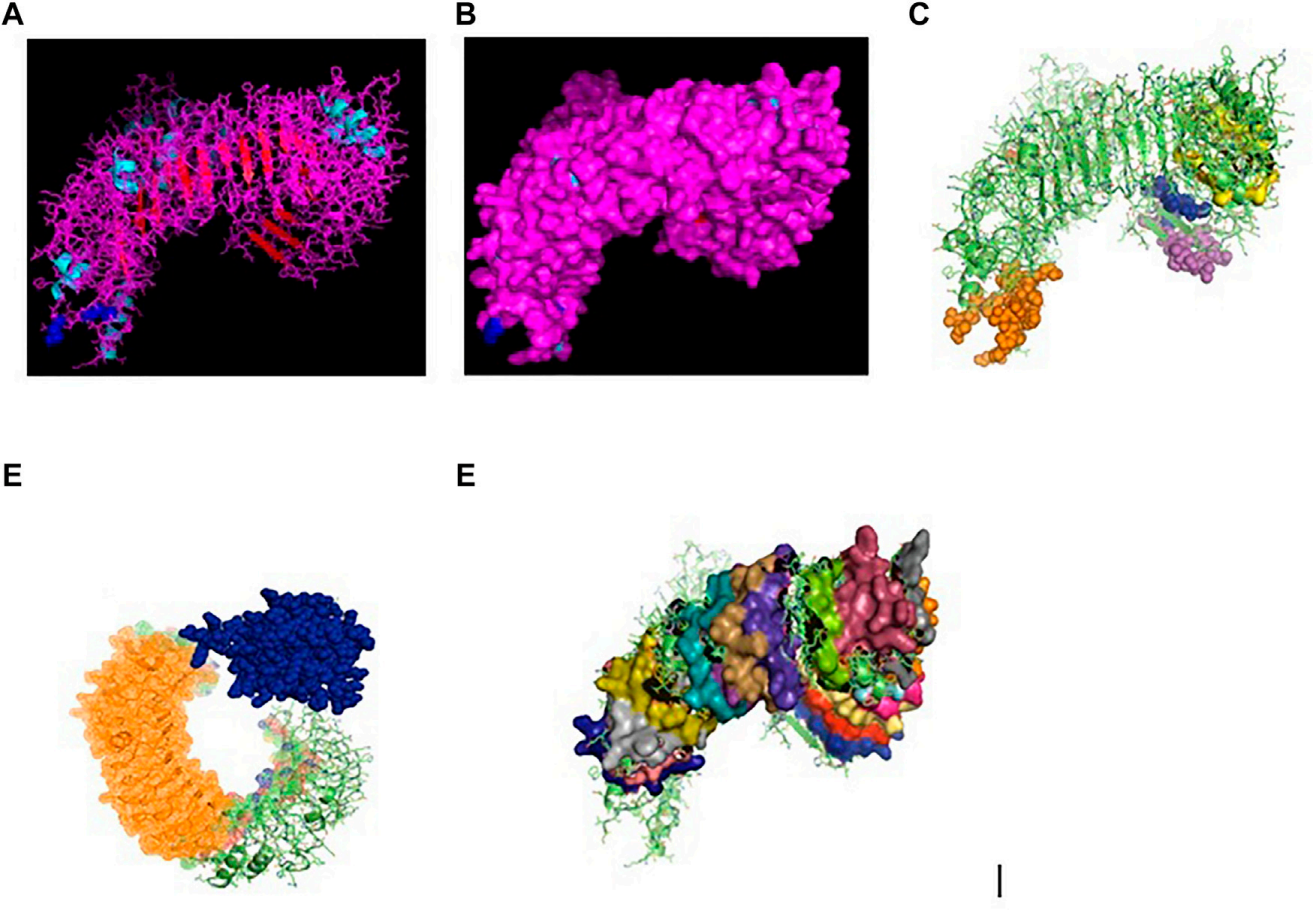
**(A)** TLR3 molecule of duck (secondary structure with disulphide bond) as blue sphere. **(B)** TLR3 molecule of duck (secondary structure with disulphide bond) surface view. **(C)** 3D structure of TLR3 of duck: yellow surface: leucine zipper (151–172), red sphere: GPI anchor (879), blue sphere: leucine-rich nuclear export signal, magenta sphere: LRRNT (37–51), orange sphere: LRRCT (664–687). **(D)** 3D structure of TLR3 of indigenous duck, blue sphere: TIR (748–890), orange mesh: leucine-rich receptor-like proteinkinase. **(E)** 3D structure of TLR3 of duck with leucine-rich repeat.

Toll-like receptors are a group of pattern recognition receptors effective against a wide range of pathogens. TLR3 gene of indigenous ducks has been characterized with 2688 bp nucleotide (Gene bank accession number KX865107, NCBI) and derived protein as ASW23003.1. The 3D protein structure (Figure 1A) with surface view (Figure 1B) has been depicted, with helix light blue, sheet red, loop pink, and blue spheres as disulfide bonds.

Posttranslational modification sites for TLR3 of duck have been depicted in Figures 1C–E. Figure 1C reveals the 3D structure of TLR3 of duck with the sites for leucine zipper (151–172 amino acid position, yellow surface), GPI anchor (aa position 879, red sphere), leucine-rich nuclear export signal (aa position 75–83, blue sphere), LRRNT (aa position 37–51, magenta sphere), and LRRCT (aa position 664–687, orange sphere). Figure 1D depicts the 3D structure of TLR3 of duck with the site for TIR (amino acid position 748–890, blue sphere) and the sites for leucine-rich receptor–like protein kinase (amino acid position 314–637, orange mesh). Figure 1E represents the sites for leucine-rich repeats as spheres at aa sites 53–74 (blue), 77–98 (red), 101–122 (yellow-orange), 125–145 (hot pink), 148–168 (cyan), 172–195 (orange), 198–219 (gray), 275–296 (raspberry), 299–319 (split pea), 346–367 (purple-blue), 370–393 (sand), 422–444 (violet), 447–468 (deep teal), 497–518 (olive), 521–542 (green), 553–574 (gray), 577–598 (salmon), and 601–622 (density). The other sites for posttranslational modification as observed were 16 sites for N-linked glycosylation, 8 sites for casein kinase 2 phosphorylation, 8 sites for myristoylation, and 9 sites for phosphokinase phosphorylation.

In comparison for TLR3 among the avian species, 51 amino acid variations were observed, which contribute to various important domains of TLR3, including LRR, LRRCT, and TIR domains (Table 2).

**TABLE 2:** Amino acid variations for TLR3 gene in duck with other poultry species.

**Sl no.**	**Position**	**Duck**	**Chicken**	**Turkey**	**Goose**	**Domain**
1	42	K	E	K	K	LRRNT
2	61	H	L	H	H	LRR1
3	68	C	V	V	C	LRR1
4	69	H	P	P	P	LRR1
5	70	A	E	E	A	LRR1
6	74	R	Q	E	K	LRR1
7	77	K	N	N	K	LRR2
8	92	Q	K	Q	Q	LRR2
9	94	E	O	E	E	LRR2
10	106	V	K	K	V	LRR3
11	119	A	V	V	T	LRR3
12	137	D	E	E	D	LRR4
13	166	L	L	L	W	LRR5
14	179	C	Y	Y	C	LLR6
15	187	K	N	K	K	LLR6
16	192	S	K	K	S	LLR6
17	200	N	N	N	K	LLR7
18	212	F	V	F	F	LRR7
19	213	H	Q	H	H	LRR7
20	285	Y	S	S	S	LRR8
21	299	N	K	N	N	LRR9
22	306	K	E	E	K	LRR9
24	310	S	I	I	I	LRR9
28	317	S	L	L	S	LRR9
29	319	Y	Y	Y	H	LRR9
30	346	Y	H	Y	Y	LRR10
31	355	N	N	H	N	LRR10
32	360	R	R	Q	R	LRR10
33	370	N	K	K	N	LRR11
34	378	S	Y	Y	S	LRR11
35	382	I	T	I	I	LRR11
36	390	T	K	K	T	LRR11
37	423	H	Q	Q	H	LRR12
38	435	S	N	N	S	LRR12
39	444	K	E	E	K	LRR12
40	468	S	I	I	S	LRR13
41	497	Q	R	R	Q	LRR14
42	521	H	H	Y	H	LRR15
43	522	K	E	K	K	LRR15
44	539	H	C	Q	H	LRR15
45	571	Q	H	H	Q	LRR
46	577	F	H	Q	F	LRR
47	581	Y	D	N	Y	LRR
48	601	T	T	N	T	LRR
49	619	E	N	D	V	LRR
50	686	V	A	A	A	LRRCT
51	766	I	T	T	I	TIR1

### Molecular Characterization of RIGI of Duck

**FIGURE 2:**
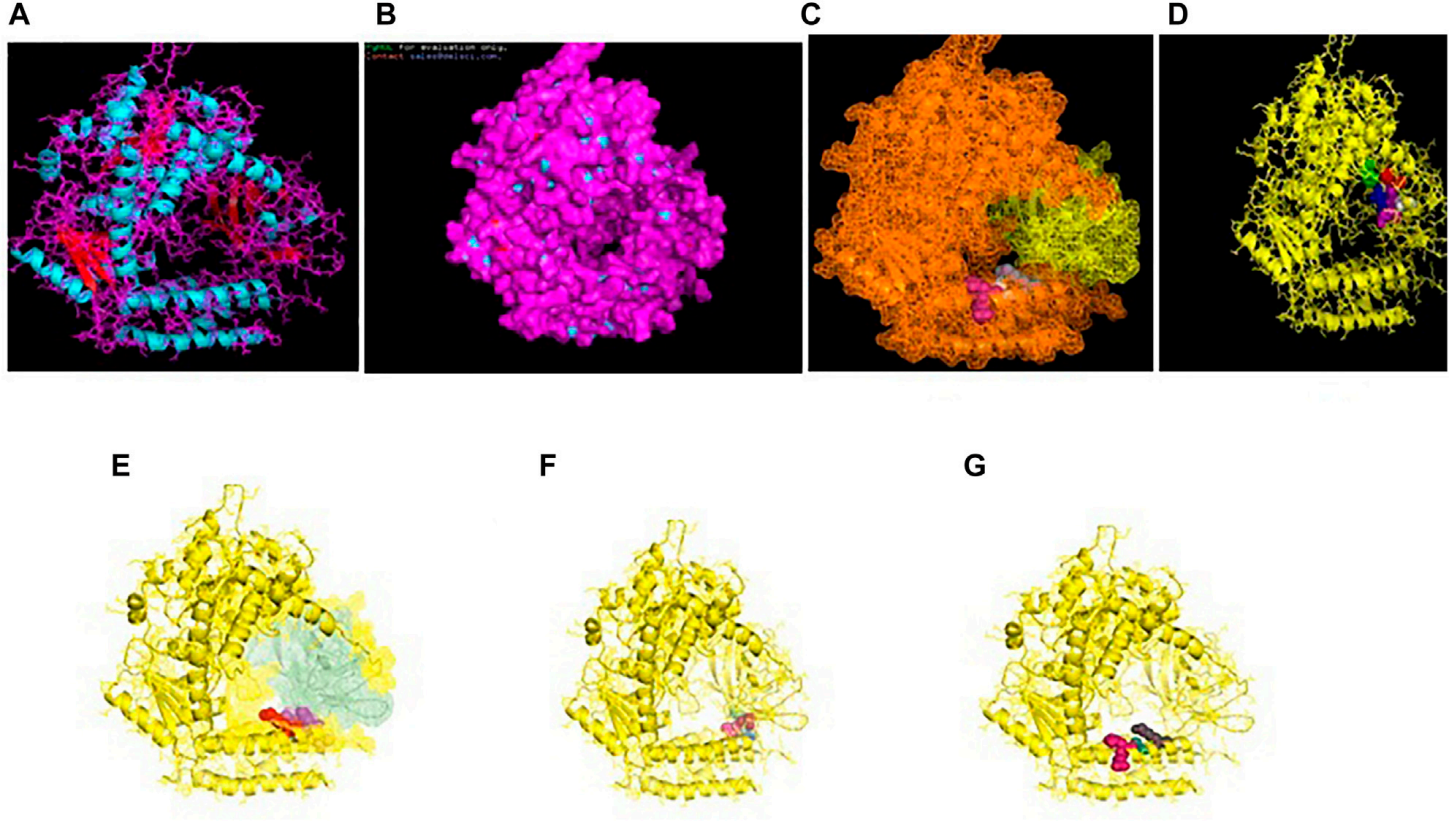
**(A)** 3D structure of RIG1 of duck. **(B)** 3D structure of RIG1 of duck, surface view. **(C)** 3D structure of RIG1 of duck with sites for helicase insert domain, helicase domain interface (polypeptide binding). **(D)** 3D structure of RIG1 of duck depicting dsRNA binding site. **(E)** 3D structure of RIGI of duck with RD interface and RIG IC. **(F)** 3D structure of RIG1 of duck for zinc binding site. **(G)** 3D structure of RIG1 of duck of RNA binding site.

RIGI of duck has been characterized (Gene bank accession number KX865107, protein ASW23002 from NCBI). The 3D structure of RIGI of duck is depicted in Figures 2A,B (surface view). RIGI is an important gene conferring antiviral immunity. A series of posttranslational modification and various domains for its important function have been represented.

**FIGURE 3:**
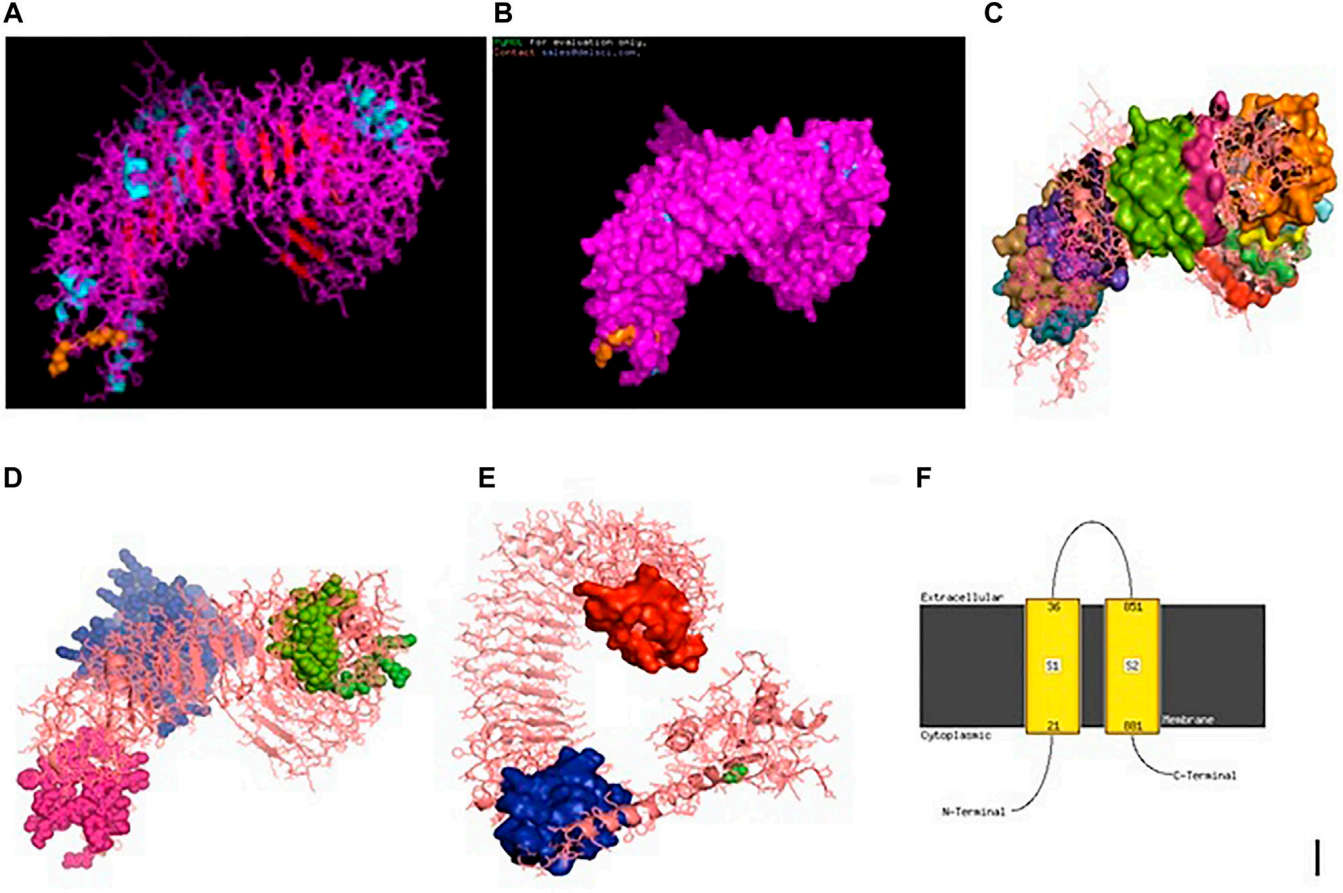
**(A)** 3D structure of TLR7 of indigenous duck. **(B)** 3D structure of TLR7 of duck (surface view). **(C)** 3D structure of TLR7 of duck with the LRR region. **(D)** 3D structure for TLR7 of duck domains. **(E)** 3D structure of TLR7 of duck with LRRNT, TPKRC2, and GPI anchor. **(F)** Transmembrane helix for TLR7 of duck.

CARD_RIG1 (caspase activation and recruitment domain found in RIG1) have been depicted in amino acid positions 2–91 and 99–188. CARD2 interaction site (17–20, 23–24, 49–50, and 79–84), CARD1 interface (100, 103, 130–135, 155, 159, and 161–162), and helical insert domain interface (101, 104–105, 107–108, 110–112, 114–115, 139, 143–145, 147–148, 151, 180, 183–184, and 186 aa) have been depicted at RIG1 of duck. Figure 3C depicts helicase insert domain (242–800 aa) as orange mesh, helicase domain interface (polypeptide binding) as (511–512aa warm pink, 515aa white, 519aa gray). Figure 2D depicts the double-stranded RNA binding site (nucleotide-binding) at amino acid positions 832 (red), 855 (green), 876–877 (blue), 889–891 (magenta), and 911 (white). The sites for RD interface (polypeptide binding) and RIG-I-C (C terminal domain of retinoic acid-inducible gene, RIG-I protein, a cytoplasmic viral RNA receptor) have been depicted in Figure 2E. The site for RIG-I-C as amino acid position 807–921 is represented by a mesh of pale green tints. The sites for RD interface have been depicted as amino acid positions 519 (red sphere), 522–523 (magenta), 536–537 (orange), and 540 (gray).

Figure 2F depicts the sites for the zinc-binding domain of RIG1 of duck as amino acid positions 812 (firebrick), 815 (marine blue), 866 (green), and 871 (hot pink). The sites for RNA binding have been depicted as 511–512 (hot pink), 515 (cyan-deep teal), and 519 (gray) in Figure 2G.

### Molecular Characterization of TLR7 of Duck

TLR7 gene has been characterized in duck (Gene bank Accession no. MK986726, NCBI). The 3D structure of TLR7 is depicted in Figures 3A,B (surface view). TLR7 is rich in leucine-rich repeat (LRR) as depicted in Figure 3C. The LRR sites are 104–125 (red sphere), 166–187 (green), 188–210 (blue), 243–264 (yellow), 265–285 (magenta), 288–309 (cyan), 328–400 (orange), 435–455, 459–480 (gray), 534–555 (warm pink), 558–628 (split pea), 691–712 (purple-blue), 715–762 (sand), and 764–824 (deep teal).

The other domains are GPI anchor at 1072 amino acid position (red sphere), domain linker sites such as 294–317 (green sphere) and 467–493 (split pea sphere) (Figure 3D). The TIR site had been identified as 929–1076 amino acid position (blue sphere) and cysteine-rich flanking region, and C-terminal as 823–874 amino acid position (hot pink) as depicted. The site for LRRNT (leucine-rich repeat N-terminal domain) of TLR7 had been identified at amino acid position 75–107 (red surface), TPKR-C2 (tyrosine-protein kinase receptor C2 Ig-like domain) at amino acid position 823–869 (blue surface) and GPI anchor as a green sphere (Figure 3E). Figure 3F represents the transmembrane site for TLR7 of duck.

### Molecular Docking of TLR3, RIGI, and TLR7 Peptides With the Antigenic Binding Sites of H and N Antigens of Avian Influenza Virus

**FIGURE 4:**
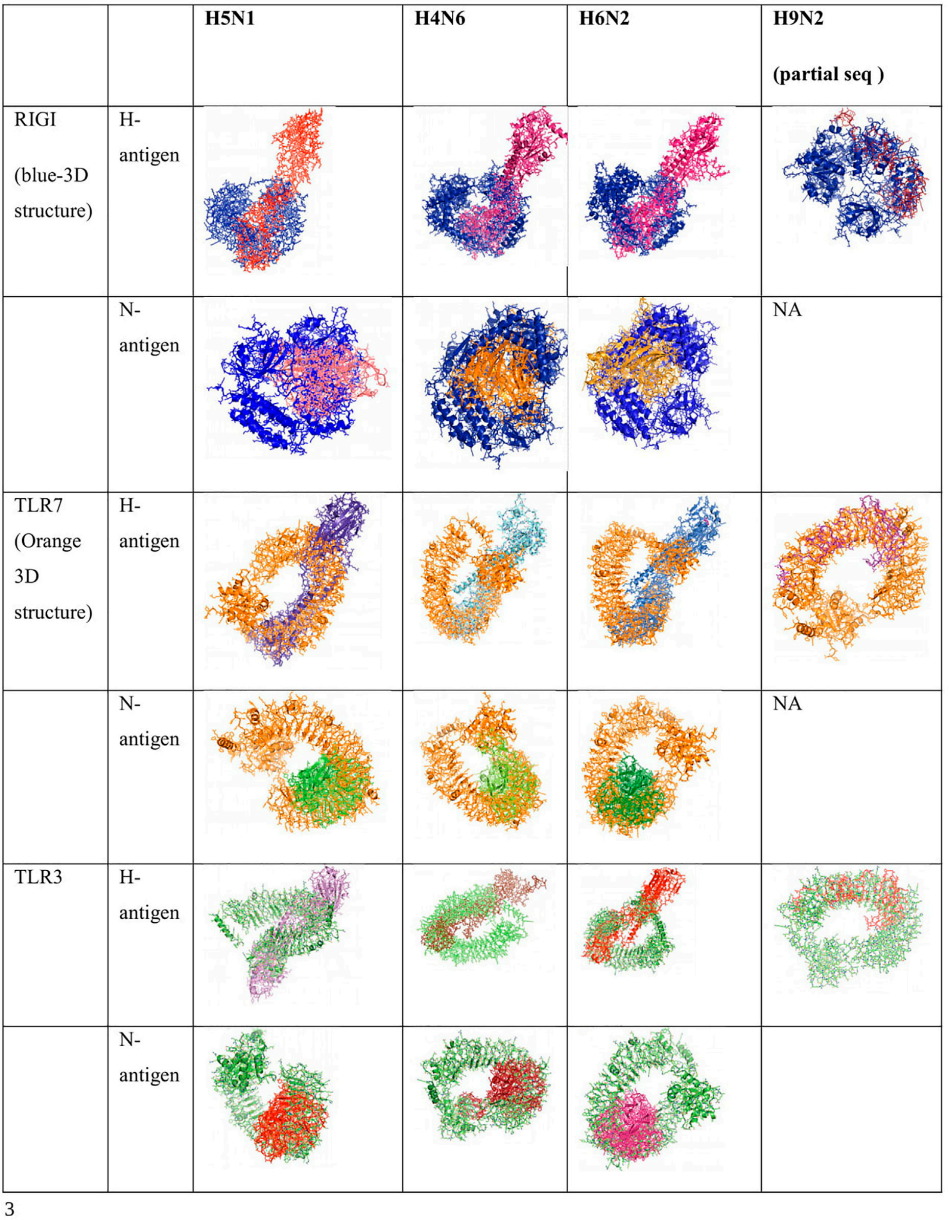
Alignment/binding of identified immune response molecules with hemagglutinin and neuraminidase for different strains of avian influenza.

Binding for H and N antigens was observed for different strains of avian influenza with RIGI, TLR7, and TLR3 (Figure 4). PatchDock analysis has revealed a high score for hemagglutinin and neuraminidase antigen for the H5N1 strain of avian influenza virus. The PatchDock score for H antigen for RIGI, TLR7, and TLR3 was observed to be 19920, 20532, and 22880, respectively, whereas the PatchDock score for N antigen for RIGI, TLR7, and TLR3 was 21570, 20600, and 21120, respectively, as detailed in Supplementary Figure S1. The binding scores were observed to be sufficiently high. The highest score was obtained for H antigen with TLR3.

Ligand binding is very much important for the receptor molecule. In our current study, we had studied only the surface antigens such as hemagglutinin and neuraminidase that are involved in binding with the immune molecules.

**FIGURE 5:**
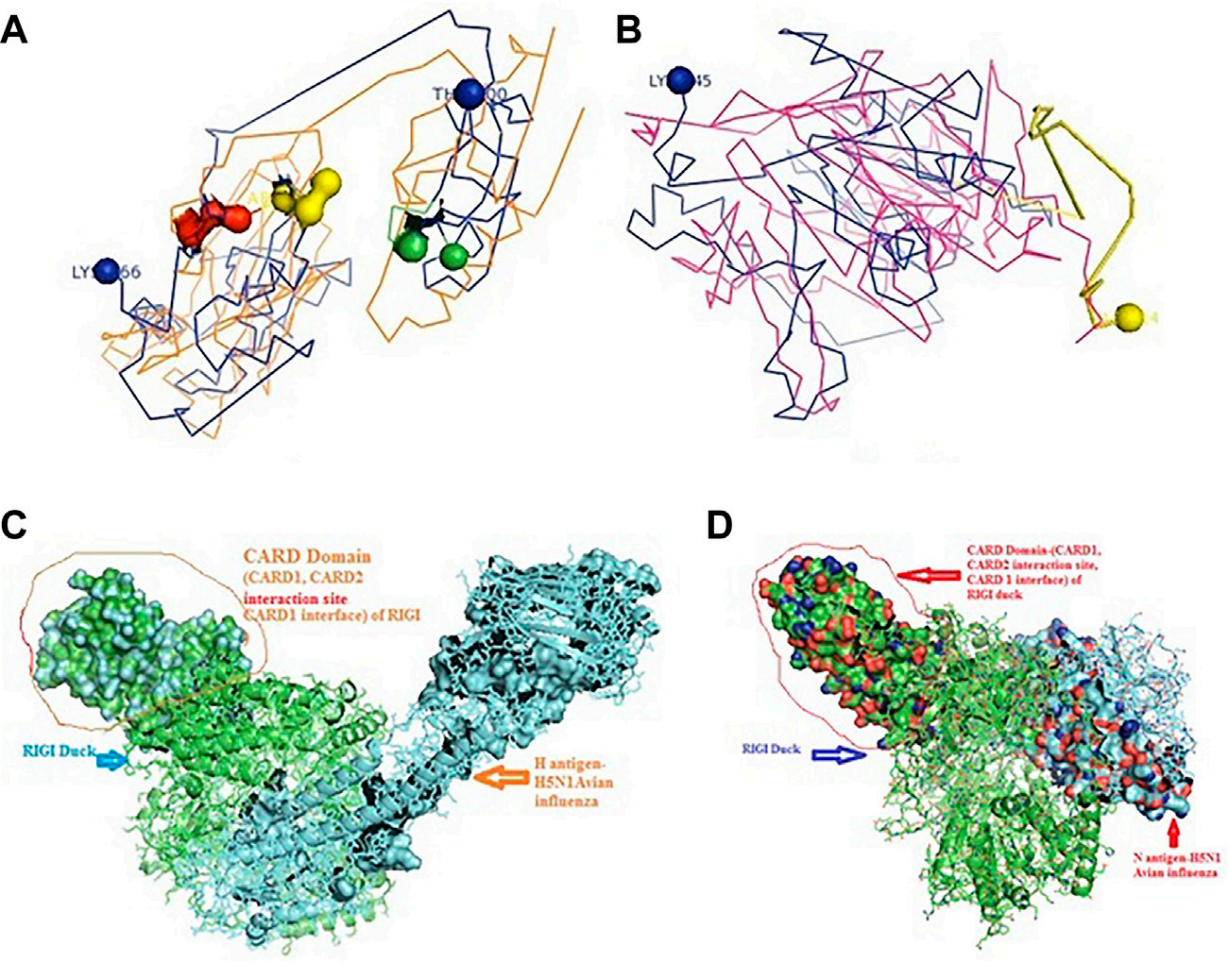
Molecular docking of RIGI with surface antigen for avian influenza. **(A)** Molecular docking image RIG1 of duck with antigen (AI) ligand H antigen-binding site detection. **(B)** Molecular docking image RIG1 of duck with antigen (AI) NS antigen ligand **(left)** and binding site **(right)**. **(C)** Duck RIGI model binding with H-antigen of avian influenza virus, with CARD domain—no binding for CARD with virus. **(D)** Duck RIGI model binding with N-antigen of avian influenza virus, with CARD domain—no binding for CARD with virus.

The binding of RIGI of duck with the hemagglutinin H5N1 strain of avian influenza is being depicted with certain domains highlighted. The binding site of RIGI with H antigen of the H5N1 strain of avian influenza virus extends from 466 to 900 amino acid positions as blue spheres (Figure 5A). The red surface indicates the helicase interface domain (511–512, 515, 519 aa position of RIGI). Amino acid position 519 is a predicted site for helicase interface domain as well as a site for RD interface. The site for helicase interface domain is depicted as the yellow surface. Another important domain within the site includes zinc binding domain depicted as the green surface.

The binding of RIGI of duck with the neuraminidase H5N1 strain of avian influenza is being depicted with certain domains highlighted. The binding site of RIGI with the N antigen of the H5N1 strain of avian influenza virus extends from lysine 245 to isoleucine 914 amino acid positions as blue spheres (Figure 5B). Figure 5B depicts only the aligned region of neuraminidase and RIGI. The site for RIG-I-C (C terminal domain of retinoic acid-inducible gene ranging from 807–921 aa position by yellow stick).

An interesting observation was that in the CARD domains such as CARD_RIG1 (caspase activation and recruitment domain found in RIG1) and CARD2 interaction site, CARD1 interface was not involved in binding with both the surface protein hemagglutinin and neuraminidase of avian influenza virus. This was proved through the pdb structure of RIGI developed with Modeller software (Figures 5C,D), respectively, for H- and N-antigen.

**FIGURE 6:**
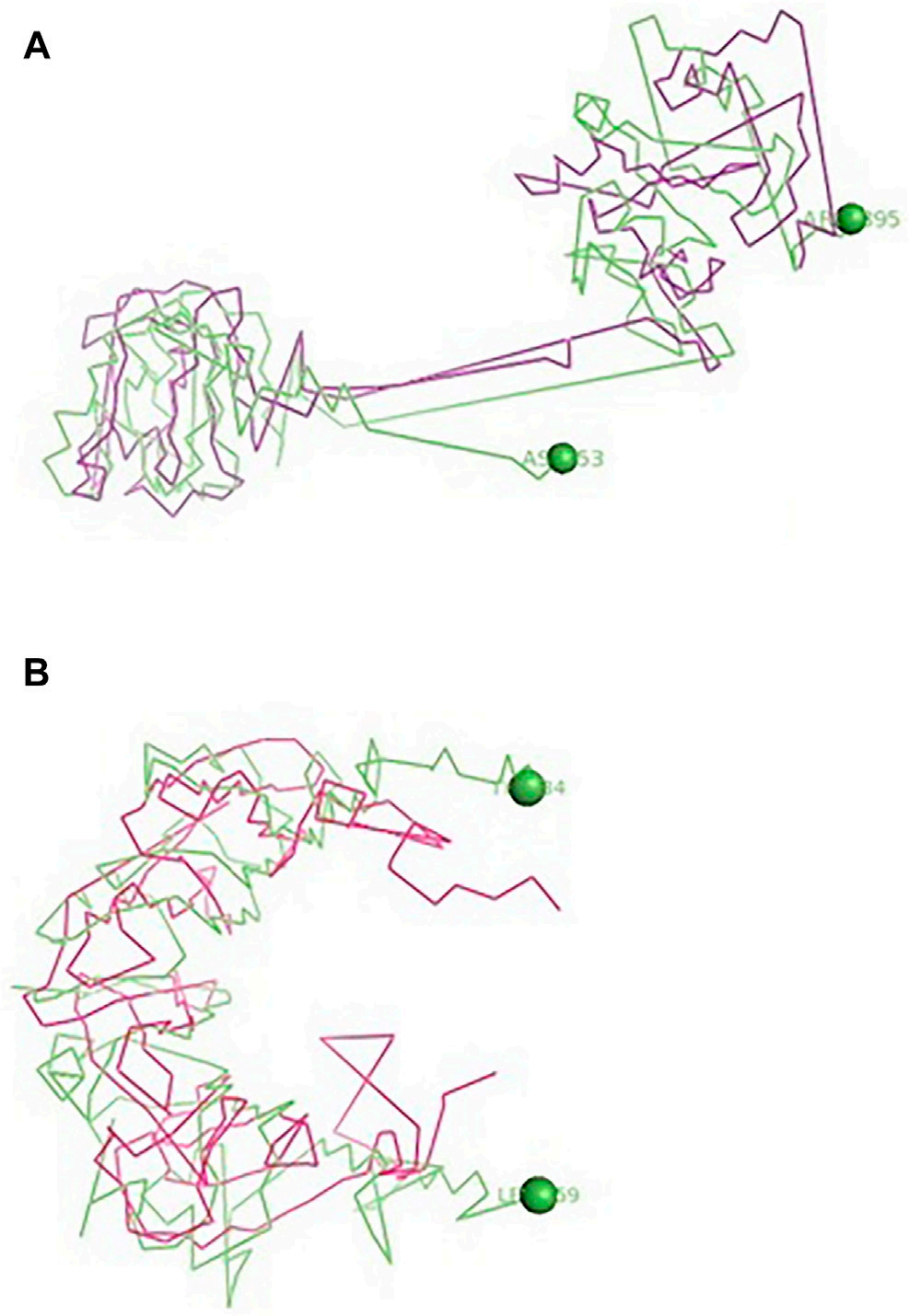
**(A)** Molecular docking -TLR3 of duck with H antigen of Avian influenza **(B)** Molecular docking TLR3 of duck with NS antigen of Avian influenza.

The binding of TLR3 of duck with the neuraminidase H5N1 strain of avian influenza is being depicted with certain domains highlighted. The binding site of TLR3 with N antigen of the H5N1 strain of avian influenza virus extends from threonine 34 to isoleucine 459 amino acid positions as green spheres (Figure 6A). Identifiable domains within this region include LLR 1 to 12, site for leucine zipper, leucine-rich nuclear export signal, and LRRNT. The domains within the 3D structure of TLR3 have been visualized already in Figures 1A–E.

The binding of TLR3 of duck with the hemagglutinin H5N1 strain of avian influenza is being depicted with certain domains highlighted. The binding site of TLR3 with the H antigen of the H5N1 strain of avian influenza virus extends from asparagine 53 to arginine 895 amino acid positions as green spheres (Figure 6B). The important domains within this region include LRR region 1–18, site for leucine zipper, GPI anchor, leucine-rich nuclear export signal, LRRCT, site for TIR, and the sites for leucine-rich receptor-like protein kinase.

**FIGURE 7:**
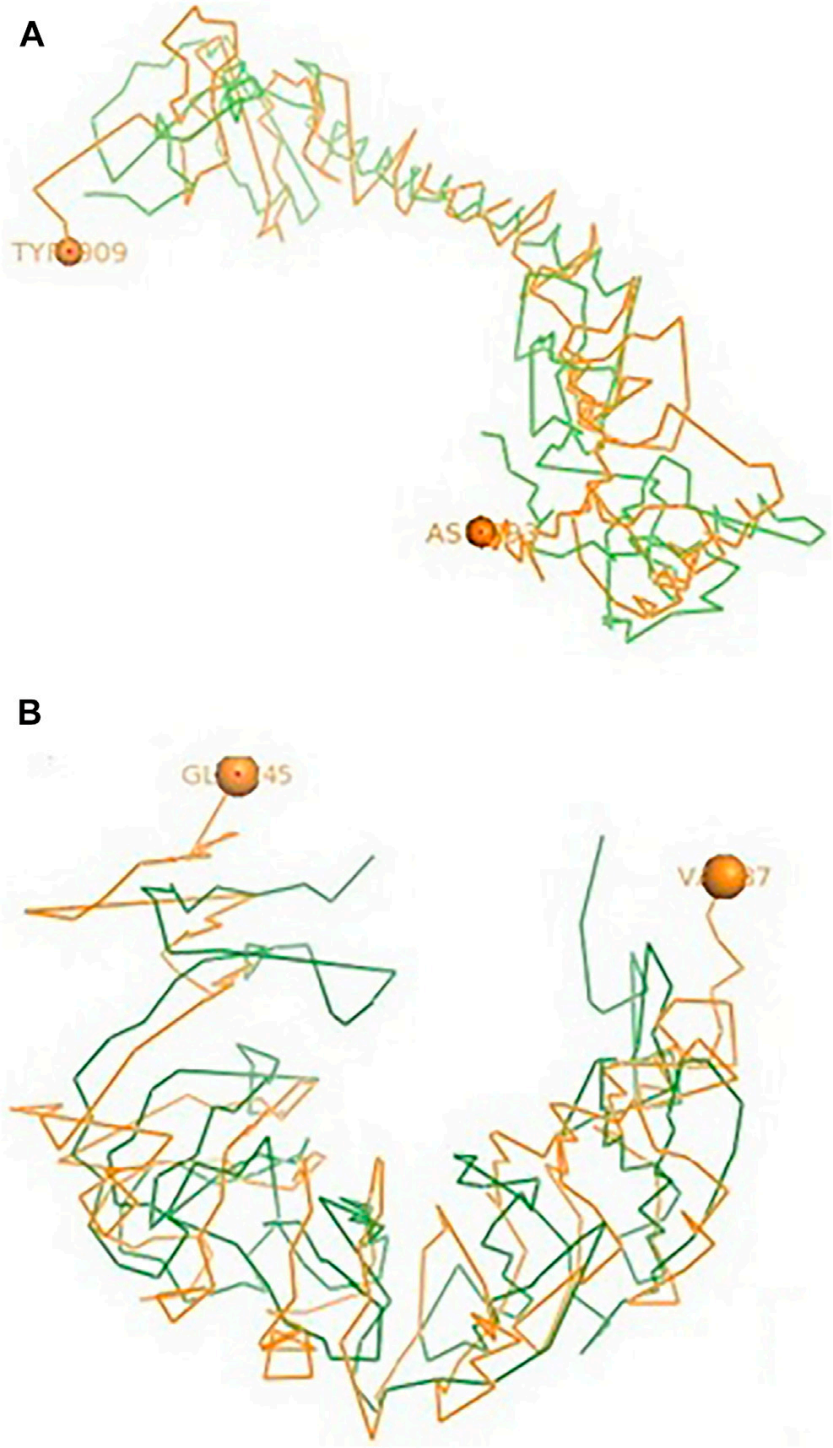
**(A)** Molecular docking TLR7 of duck with H antigen of avian influenza. **(B)** Molecular docking TLR7 of duck with NS antigen of avian influenza.

The binding of TLR7 of duck with the neuraminidase H5N1 strain of avian influenza is being depicted with certain domains highlighted. The binding site of TLR7 with the N antigen of the H5N1 strain of avian influenza virus extends from valine 87 to glutamine 645 amino acid positions as orange spheres (Figure 7A). Identifiable important domains within this region include LRR region 1–11 and domain linker sites. The detail visualization of these domains is present in Figures 3A–F in the molecular visualization tool.

The binding of TLR7 of duck with the hemagglutinin H5N1 strain of avian influenza is being depicted with certain domains highlighted. The binding site of TLR7 with the H antigen of the H5N1 strain of avian influenza virus extends from aspartic acid 293 to tyrosine 909 amino acid positions as orange spheres (Figure 7B). The important domains responsible within this binding site include LRR 7 to LRR14, domain linker sites, and cysteine-rich flanking region C-terminal, and TPKR-C2 (tyrosine-protein kinase receptor C2 Ig like domain).

### Amino Acid Sequence Variability and Molecular Phylogeny Among Different Strains of Avian Influenza

**FIGURE 8:**
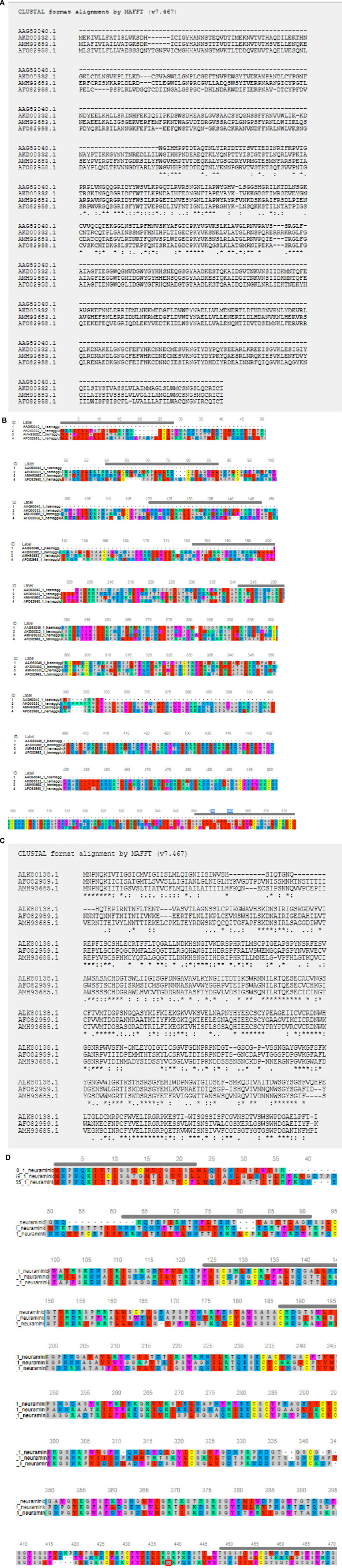
**(A)** Alignment report for Haemagglutinin antigen for different strains for Avian influenza **(B)** Alignment report for Haemagglutinin antigen for different strains for Avian influenza by MAFFT software (MSA viewer) **(C)** Alignment report for neuraminidase antigen for different strains for Avian influenza **(D)** Alignment report for neuraminidase antigen for different strains for Avian influenza by MAFFT software (MSA viewer).

High degree of sequence variability has been observed in Figures 8A,B in the hemagglutinin segment of avian influenza and Figures 8C,D in the neuraminidase segment of avian influenza. The H5N1 strain was observed to be clustered with the H6N2 strain of avian influenza virus. The H4N6 strain which is comparatively less virulent and hence causing LPAI was found to possess certain uniqueness in amino acid sequence. Deletions of four consecutive amino acids at positions 65–68 and 140–141 were observed in hemagglutinin. Likewise, insertion mutations were also observed at amino acid positions 19–24 and 77–79 in hemagglutinin. Cysteine residues were observed to be conserved across the strains (Figure 8B).

However, in such a case, highly pathogenic H5N1 depicts certain deletion mutations at amino acid positions 37–45, 53–63, and 80–82 of neuraminidase (Figure 8D).

### Comparative Structural Analysis of TLR3 and TLR7 of Duck With Respect to Chicken

**FIGURE 9:**
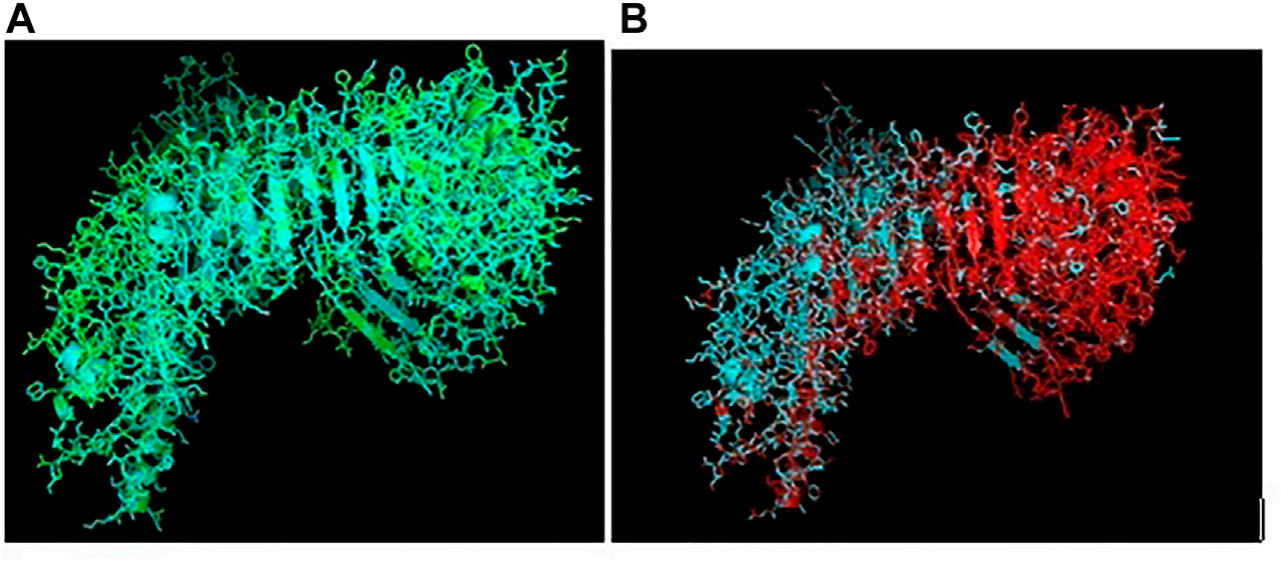
**(A)** Comparison of TLR3 of duck and chicken-3D structural analysis **(B)** Comparison of TLR7 of duck and chicken-3D structural analysis.

Ducks were reported to be genetically more resistant to chicken, particularly in terms of viral infections. Accordingly, the structural alignment of the 3D structure of TLR3 of duck with chicken has been described (Figure 9A). 3D structural alignment of TLR7 of duck with chicken has been visualized (Figure 9B). It was not possible to study the structural alignment of duck RIG-I since RIGI was not expressed in chicken.

The sites for non-synonymous mutations have been depicted for TLR3 gene in duck with respect to other poultry species such as chicken, goose, and guineafowl (Table 2). 51 sites for amino acid substitutions have been detected, ranging from amino acid position 42 to 766 in duck with respect to other poultry species, which actually contribute to changes in functional domains of TLR3. A comparison of TLR3 of duck with chicken actually revealed 46 sites of amino acid substitution resulting due to non-synonymous mutations, which are of much importance to our present study. Most of the substitutions caused changes in leucine-rich repeats, which is an inherent characteristic for pattern recognition receptor such as TLR2. 20 sites of amino acid substitutions that were specific for anseroides (duck and goose) were identified.

### Protein–Protein Interaction Network Depiction for TLR3 and TLR7 With Respect to Other Functional Proteins

**FIGURE 10:**
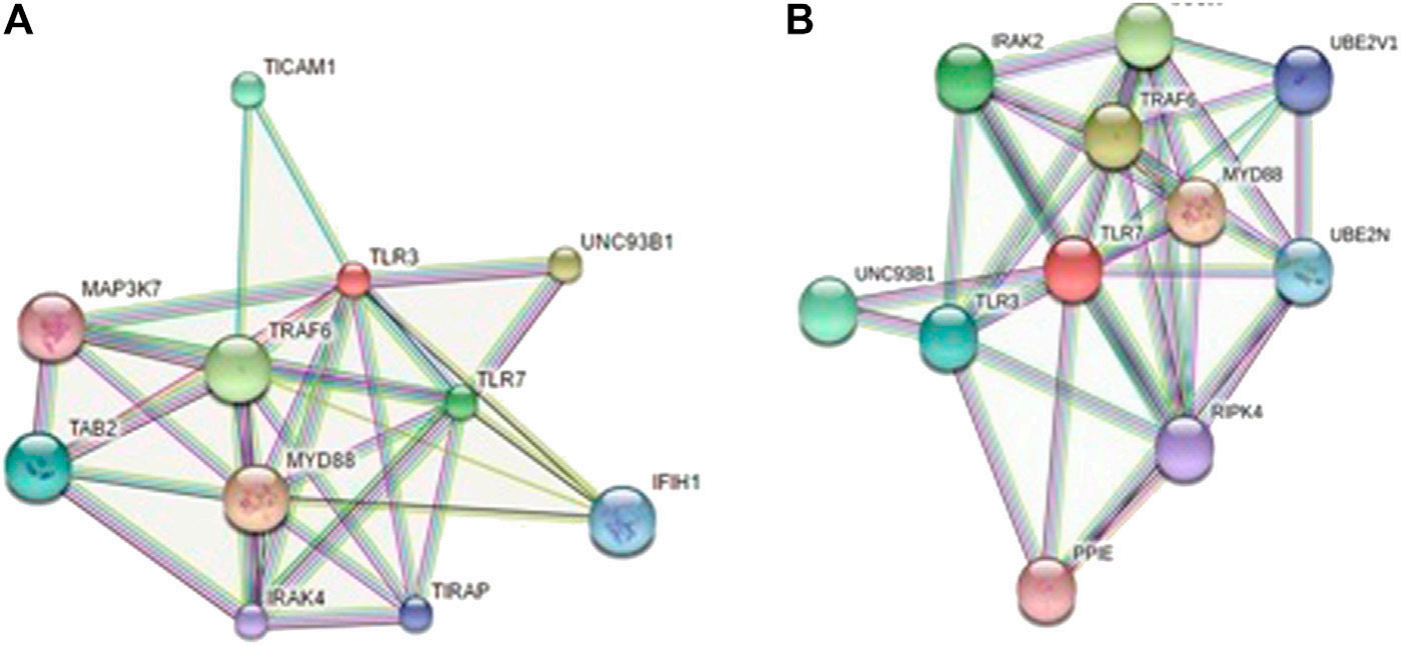
**(A)** String analysis revealing molecular interaction of TLR3 **(B)** String analysis revealing molecular interaction of TLR7.

Interaction of TLR3 with other proteins has been depicted in Figure 10A with STRING analysis. Interaction of TLR7 with other proteins of functional interest has been depicted in Figure 10B. KEGG analysis depicts a mode of the defense mechanism of influenza A and the possible role of antiviral molecules in combating the infection and the role of antiviral molecules through the TLR signaling pathway.

### Differential mRNA Expression Pattern of TLR7 and Other TLR Genes of Duck With Respect to Chicken and Other Poultry Species

**FIGURE 11:**
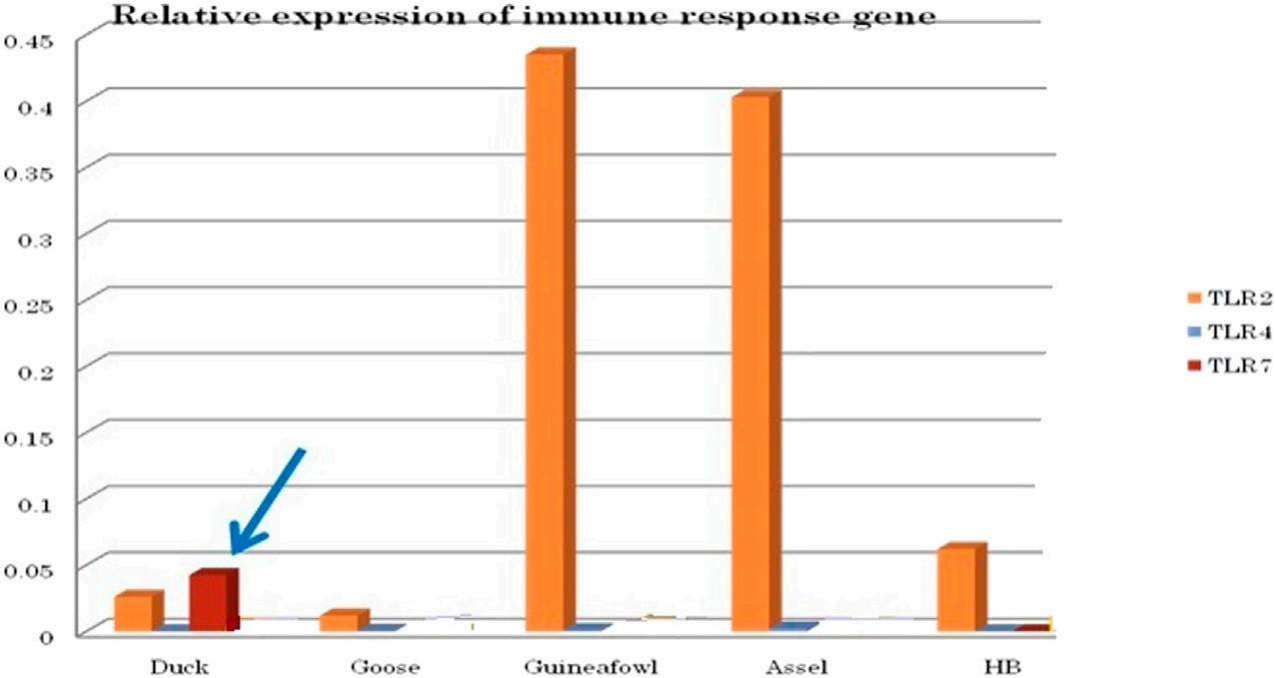
Differential mRNA expression profile of immune response genes of healthy duck with other poultry species.

We conducted differential mRNA expression profiling of TLR2, TLR4, and TLR7. Expression profiling of TLR2 and TLR4 was observed to be better in indigenous chicken (Aseel and Haringhata Black) and guineafowl than in anseroides (duck and guineafowl). Both TLR2 and TLR4 are known to impart antibacterial immunity. Quantitative mRNA expression analysis clearly depicts TLR7 gene expression was definitely better in duck than in other poultry species such as goose, guineafowl, and indigenous chicken breed (Aseel and Haringhata Black chicken) (Figure 11). This gives an indication that better immune response of indigenous duck may be due to the increased expression level of TLR7, which confers antiviral resistance (Figure 11).

**FIGURE 12:**
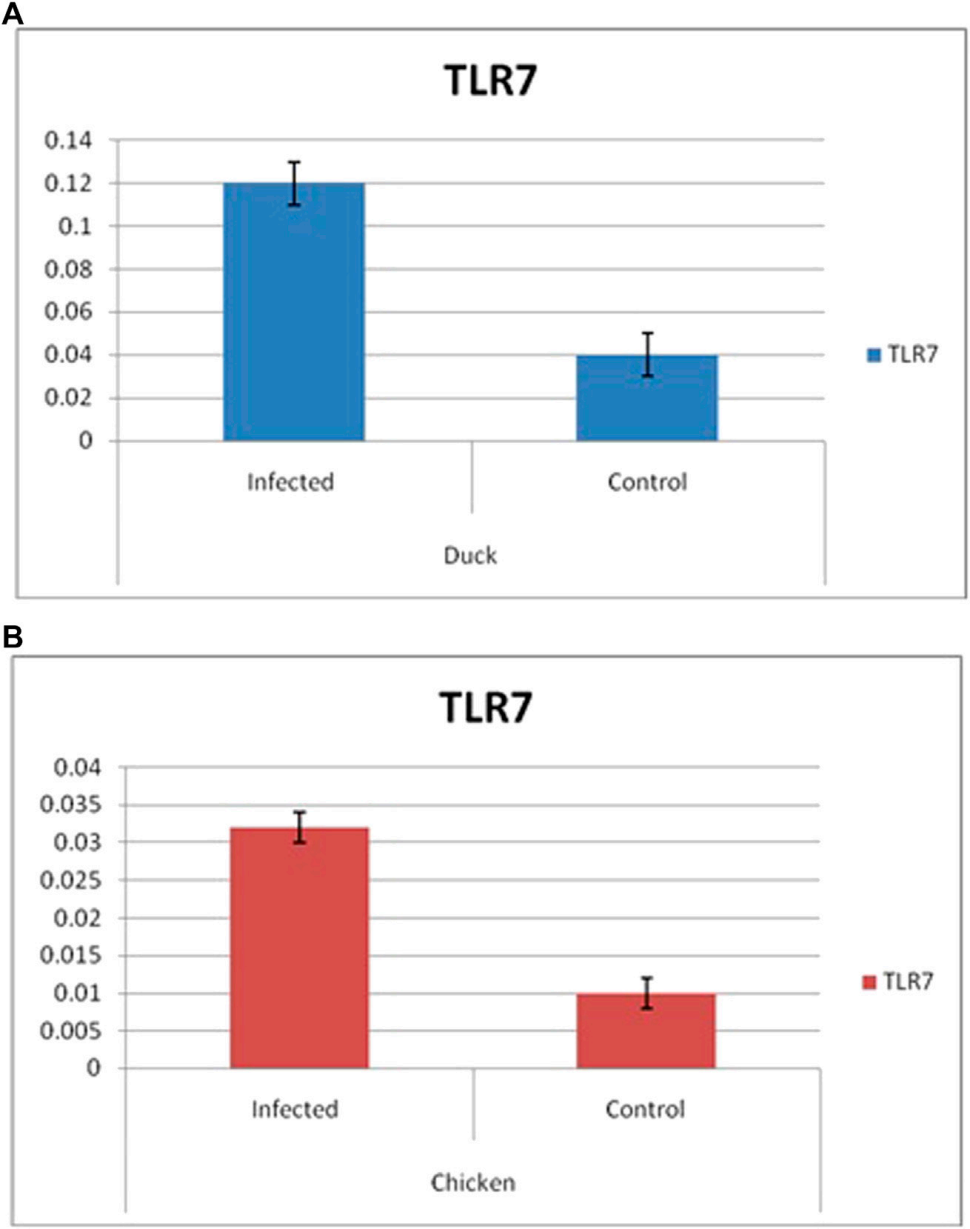
Differential mRNA expression profiling for TLR7 gene with respect to infected versus control Bengal duck and HB Chicken.

**FIGURE 13:**
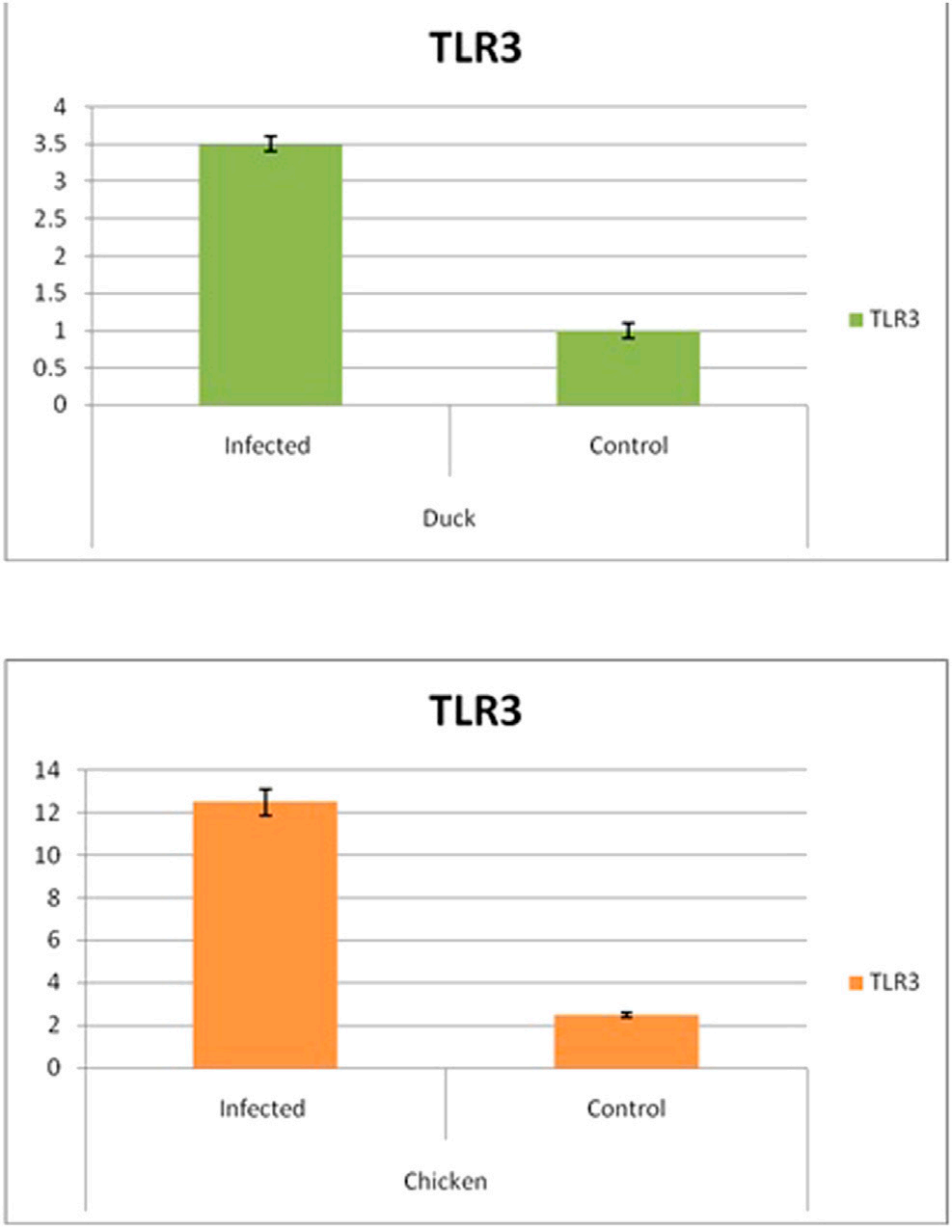
Differential mRNA expression profiling for TLR3 with respect to infected versus control Bengal duck and HB Chicken.

**FIGURE 14:**
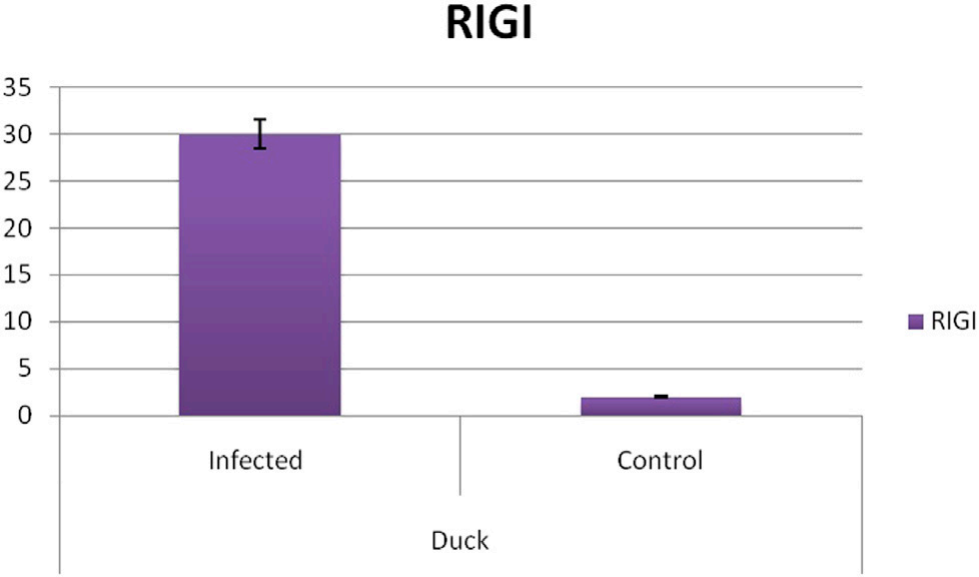
Differential mRNA expression profiling for RIGI gene with respect to infected versus control Bengal duck and HB Chicken.

Differential mRNA expression profiling for TLR7, TLR3, and RIGI genes with respect to infected versus control Bengal duck and HB chicken have been depicted in Figures 12–14, respectively. Figures 12A,B depict the TLR7 gene expression level in the infected condition with respect to healthy control in duck and chicken, respectively. Similarly, Figures 13A,B represent the TLR3 gene expression level in the infected condition with respect to healthy control in duck and chicken, respectively. However, Figure 14 represents only the expression of RIGI in duck. In all the cases, the higher expression level of TLR7, TLR7, and RIGI was detected in infected cases than in healthy control birds. RIGI was reported to have significantly pronounced better expression in infected ducks than healthy control.

### Phylogenetic Analysis of Indigenous Ducks With Other Poultry Species and Other Duck Population Globally

**FIGURE 15:**
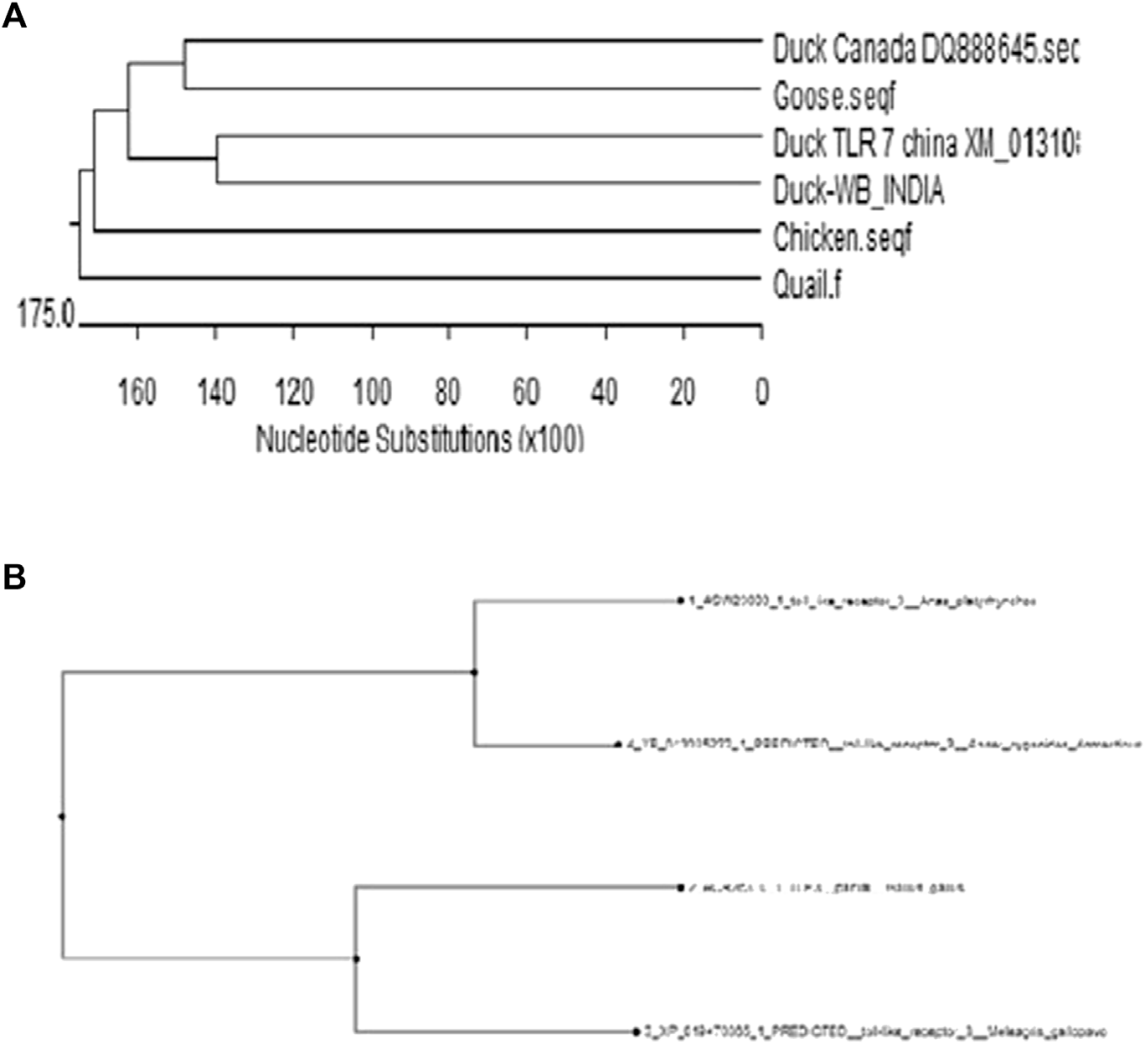
**(A)** Molecular evolution of ducks reared globally in relation to other poultry species (Based on TLR7) **(B)** Molecular evolution of ducks reared globally (Based on TLR3).

With an aim for the identification of the status of molecular evolution of duck, the indigenous duck gene sequence of West Bengal, India, was compared with other duck sequences globally. Phylogenetic analysis was performed with respect to TLR7 (Figure 15A) and TLR3 (Figure 15B). Phylogenetic analysis revealed that ducks of West Bengal were observed to be genetically more closely related to the duck population of China (Figure 15A). Ducks were observed to be genetically closest to goose (Figure 15B). Chicken, quail, and turkey were observed to be genetically distinct from duck (Figure 12B).

## Discussion

Indigenous duck population was characterized to be very hardy, and usually asymptomatic to common avian diseases. But there is a paucity of information regarding the systemic genetic studies on duck involved in its unique immune status. It is evident that duck possesses some unique genetic makeup which enables it to provide innate immunity against viral infection, particularly avian influenza.

In this current study, we identified three immune response molecules, which were earlier known to have immunity against viral infection such as RIGI, TLR7, and TLR3. We characterized these proteins of duck, and attempted to identify the SNPs or variations in nucleotide among duck and chicken. Through molecular docking, the most promising IR molecules conferring innate immunity against avian influenza have been identified, which were later on confirmed through the wet lab study as differential mRNA expression profiling. We attempt to explore the unique genetic constitution of duck immune response with respect to that of chicken. However, RIGI was reported to be expressed only in duck, not in chicken; hence, comparison was not available. TLR7 expression profiling was observed to be significantly better in duck than in chicken and other poultry species, indicative of better antiviral immunity in ducks. 53 non-synonymous mutations with amino acid variations were observed while comparing amino acid sequence of duck with other poultry species, including chicken, most of which are confined to the LRR domain. We had already depicted that LRR is an important domain for pathogen binding site as in this case of avian influenza. For the effective antiviral activity, binding of viral protein with the immune response molecule is the primary criterion. Leucine-rich repeats (LRRs) were observed to be important domain involved in binding with hemagglutinin and neuraminidase surface protein in case of both TLR3 and TLR7. Similar studies have also reported LRR as the important domain against bacterial infections in case of CD14 molecule in cattle (Stetson and Medzhitov, 2006), goat (Vercammen et al., 2008a; Schlee et al., 2009), and buffalo (Pal and Chatterjee, 2009; Pal et al., 2013). Other important domains identified were LRRNT, LRRCT, site for TIR, and the sites for leucine-rich receptor-like protein kinase, including certain posttranslational modification sites. Similar reports were also identified in different species (Stetson and Medzhitov, 2006; Vercammen et al., 2008a; Pal and Chatterjee, 2009; Schlee et al., 2009; Pal et al., 2013). An important observation identified was that although the CARD domain was believed to be an important binding site for RIGI for some identified virus, it has no binding ability with avian influenza virus. Other studies have reported the role of the CARD domain in binding with the MAVS domain as a part of antiviral immunity (Kelley and Sternberg, 2009; Šali et al., 2020).

TLR3 (CD283 or cluster of differentiation 283) is a pattern recognition receptor rich in leucine-rich repeats as revealed in duck TLR3 of the current study. Other important domains include LRRNT, LRRCT, TIR, leucine-rich receptor-like protein kinase, leucine zipper, GPI anchor, and leucine-rich nuclear export signal. The other sites for posttranslational modification as observed were N-linked glycosylation, casein kinase, phosphorylation, myristoylation, and phosphokinase phosphorylation. Variability of amino acids in important domains was observed for duck TLR3 compared to that of chicken, goose, and turkey. It was observed that genetic similarity between duck and goose was more than that of chicken and turkey. Some amino acids which are conserved for ducks and denote for important domains such as LRR, LRRCT, and TIR have been identified. In the LRRCT domain, valine is present in a duck in contrast to alanine in chicken, turkey, and goose. The current study identified 45 sites of non-synonymous substitutions between duck and chicken, which affect important domains for TLR3 as a pattern recognition receptor. Although it is the first report of characterization of TLR3 in indigenous duck, earlier studies were conducted in Muscovy duck, when full-length cDNA of TLR3 was characterized to be of 2836 bp encoding polypeptide of 895 amino acids (Zhang and Skolnick, 2005). The characterization of the deduced amino acid sequence contained 4 main structural domains: a signal peptide, an extracellular leucine-rich repeats domain, a transmembrane domain, and a Toll/IL-1 receptor domain (Zhang and Skolnick, 2005), which is in agreement to our current study. It is to be noted that TLR3 is a PRR, with the secondary structure being visualized as helix, loop, and sheet with the sites for disulfide bond being depicted in blue spheres. It recognizes dsRNA associated with a viral infection, and induces the activation of IRF3, unlike all other Toll-like receptors which activate NF-κB. IRF3 ultimately induces the production of type I interferons, which aid in host antiviral immunity (Morgan et al., 2019). In the current study, we observed sites for leucine zipper in duck TLR3, which is an inherent characteristic for dimerization. Earlier studies have also reported that TLR3 forms a large horseshoe shape that contacts with a neighboring horseshoe, forming a “dimer” of two horseshoes (Niu et al., 2019). As already explained that glycosylation is an important PTM (posttranslational modification site), it acts a glycoprotein. But in the proposed interface between the two horseshoe structures, two distinct patches which are rich in positively charged amino acids and may be responsible for the binding of negatively charged viral dsRNA were observed.

RIG1 [also known as DEAD-box protein 58 (DDX58)] is an important molecule conferring antiviral immunity. Various important domains have been identified, such as CARD_RIG1 (caspase activation and recruitment domain found in RIG1), CARD2 interaction site, CARD1 interface, helicase insert domain, double-stranded RNA binding site, RIG-I-C (C terminal domain of retinoic acid–inducible gene, RIG-I protein, and a cytoplasmic viral RNA receptor), RD interface, zinc-binding domain, and RNA binding. CARD proteins were observed to be responsible for the recognition of intracellular double-stranded RNA, a common constituent of a number of viral genomes. Unlike NLRs, these proteins, RIG-I contain twin N-terminal CARD domains and C-terminal RNA helicase domains that directly interact with and process the double-stranded viral RNA. CARD domains act through the interaction with the CARD motif (IPS-1/MAVS/VISA/Cardiff) which is a downstream adapter anchored in the mitochondria (Vercammen et al., 2008b; Botos et al., 2011).

Through the *in silico* alignment study, it was clearly observed that TLR3 binds with the H antigen of the avian influenza virus (H5N1). It was known that avian influenza virus (H5N1) strain is 100–200 nm spherical, enveloped, and includes 500 projecting spikes containing 80% hemagglutinin and 20% neuraminidase (Bouvier and Palese, 2008), with genome being segmented antisense ssRNA. In order to combat the infection, TLR3 binds with the hemagglutinin spikes of the influenza virus. TLR3 gene was observed to have a role to combat against Marek’s disease (Said et al., 2018): seven amino acid polymorphism sites in ChTLR3 with 6 outer part sites and 1 inner part site (Barber et al., 2010). TLR3 cannot act alone. It acts while interacting with a series of molecules such as TICAM1, MAP3K7, TAB2, TRAF6, Myd88, IRAK4, IFIH1, and even TLR7. TLR3 acts through the RIG1-like receptor signaling pathway and acts through TRIF (Said et al., 2018). Along with *in silico* studies, we validated these findings with experimental challenge with avian influenza virus in embryonated fibroblast cell. The expression was observed to be more in infected egg than in healthy control for these genes in duck and chicken.

RIG1 is an important molecule which is only expressed in ducks, not in a chicken. Duck RIGI transfected cells were observed to recognize the RIG-I ligand, and a series of antiviral genes were expressed such as IFN-β, MX1, PKR, IFIt5, and OASI, and consequently, HPAIV (highly pathogenic avian influenza virus) titers were reduced significantly (Haunshi and Cheng, 2014a; Ruan et al., 2015). RIG-I belongs to the IFN-stimulated gene family, and it acts through the RIG-I–like receptor signaling pathway. RIG1 detects dsRNA virus in the cytoplasm and initiates an antiviral response by producing type-I and Type-III IFN, through the activation of the downstream signaling cascade. RIG-I is an IFN-inducible viral sensor and is critical for amplifying the antiviral response (Potter et al., 2008; Barber et al., 2012). Although RIG1 expression is absent in chicken; it can produce INFα by another pathway. It has been observed that IFN-β expression upon influenza infection is mediated principally by RIG1 (Yoneyama et al., 2004). INFα expression induced by chicken is unable to protect the host from avian influenza infection as IFN-β, produced in ducks (Bouvier and Palese, 2008; Takahasi et al., 2008). This may be one of the major reasons why ducks are resistant to HPAIV, but not chicken. Since the RIG1 gene is not expressed in chicken, a comparative study was not possible. Avian influenza virus was observed to have surface glycoproteins as hemagglutinin and neuraminidase spikes on its outer surface (Loo et al., 2008). It was observed that duck RIG1 can bind with both H antigen and NA antigen of avian influenza virus. It is interesting to note how RIGI acts on virus and causes destruction. RIG-I acts through the RIG-I–like signaling pathway, and secretes NRLX1 and IPS1, leading to the production of IKKβ, which in turn causes the secretion of NFκβ and Iκβ (Koemer et al., 2007). These substances ultimately cause viral myocarditis and the destruction of the virus (Loo and Gale, 2011). A sequence of reactions occurs such as high fever, acute respiratory distress syndrome, chemoattraction of monocytes and macrophages, T-cell activation, and antibody response (Haunshi and Cheng, 2014b).

TLR7 is another important molecule responsible for antiviral immunity; it recognizes single-stranded RNA as the genetic material. It acts through the Toll-like receptor signaling pathway. However, from the current study, it was observed that TLR7 binds well with the NA antigen. The identified domains for TLR7 are mainly LRR (leucine-rich repeat), TIR, cysteine-rich flanking region, LRRNT (leucine-rich repeat N-terminal domain), and TPKR-C2 (tyrosine-protein kinase receptor C2 Ig-like domain). TLR7 releases Myd88, which in turn releases IRAK (Yamada et al., 2018). Ultimately, IRF7 is released, which causes viral myocarditis. It is interesting to note that in human and mice, TLR7 is alternatively spliced and expressed as two protein isoforms (Brisse and Ly, 2019). Another interesting observation was that chicken erythrocytes do not express TLR7 (Heil et al., 2003). While studying the TLR7 expression pattern in different avian species, an interesting observation in our current study was that TLR 7 gene expression was significantly better in duck than in other poultry species, such as indigenous chicken breeds (Aseel and Haringhata Black chicken), goose, and guineafowl. This is the first report for such a comparative study. It was reported that chicken TLR7 follow a restricted expression pattern. TLR7 expression was better in a macrophage cell line, chicken B-cell–like cell line, but the expression was observed to be lower in kidney cell line (Philbin et al., 2005). Following Marek’s disease virus expression, TLR7 expression was observed to be increased in the lungs (St Paul et al., 2013).

Similarly, increased TLR7 expression was noted in IBDV (infectious bursal disease virus) (Iqbal et al., 2005). With regard to avian influenza infection, it was observed that at the early stage of low pathogenic avian influenza virus (LPAIV) infection of H11N9, in both duck and chicken, TLR7 is transiently expressed in peripheral blood mononuclear cells (PBMCs), while as infection progresses, the expression declines. Hence, it was observed that in chicken, TLR7 expression depended on the interaction between host and RNA virus (Abdul-Careem et al., 2009). Thus, differences in the expression pattern of TLR7 in chicken and duck were suggested (Abdul-Careem et al., 2009). Even in chicken, the TLR7 expression pattern was found to vary between HPAIV and LPAIV. Thus, TLR7 was observed to be an important immune response gene for avian influenza; TLR7 ligands show considerable potential for antivirals in chicken (Abdul-Careem et al., 2009). Although no direct report was available for better TLR7 expression in a duck than in a chicken, it was reported that tissue tropism and immune function of duck TLR7 are different from those of chicken TLR7, which result in a difference in susceptibility between chicken and duck, when infected by the same pathogen (Abdul-Careem et al., 2009). A high expression pattern of duck TLR7 in respiratory and lymphoid tissue was observed to be different from that of chicken.

TLR3, TLR7, and TLR21 localize mainly in the ER in the steady-state and traffic to the endosome, where they engage with their ligands. The recognition triggers the downstream signal transduction to activate NF-κв or IRF3/7, finally induces interferon and inflammatory cytokine production (Abdul-Careem et al., 2009). We can explore these identified and characterized genes for production of transgenic or gene-edited chicken resistant to avian influenza as a future control strategy against avian influenza through immunomodulation, devoid of side effects as in case of use of drugs (Rauf et al., 2011). It is to be noted that since the control of avian influenza virus has been difficult and challenging either through vaccination (Chen et al., 2013; Rios et al., 2017; Rx List, 2019) or treatment through antiviral drugs (FDA, 2020; Science News, 2016; Principi et al., 2019; WebMD, 2020) due to frequent mutation and genetic reassortment (regarded as antigenic shift or antigenic drift) of the single stranded RNA genome which is prone to mutations (NHS, 2014; Zhang et al., 2019; Richard et al., 2002a). An interesting observation revealed that unlike antibodies (comprising of immunoglobulins) which were highly specific, arising due to variability of Fab site and variable region (Nguyen, 2020), immune response molecules for innate immunity can bind Avian influenza virus (H, N antigen), irrespective of strains. As we analyze the binding sites, some important domains were identified, which may be involved in antiviral activity. This led to the finding that the therapeutic approach may be attempted with the recombinant product corresponding to the identified domain. Gene editing with gene insert from identified gene may lead to the evolution of disease-resistant strains/lines of chicken or duck.

Although the receptors for human influenza and avian influenza are different, mutations may overcome the barrier. As a case report in 2018, a human infection with a novel H7N4 avian influenza virus was reported in Jiangsu, China. Circulating avian H9N2 viruses were reported to be the origin of the H7N4 internal segments, unlike both the human H5N1 and H7N9 viruses that had H9N2 backbones. The major concern is that genetic reassortment and adaptive mutation of avian influenza virus give rise to human influenza virus strain H7N4 (Jiao et al., 2012; Ayora-Talavera, 2018; Karen, 2018; Li et al., 2020). The WHO has also warned about the pandemic on human flu resulting from genetic reassortment of avian influenza (Qu et al., 2020). We observed in this study that the H5N1 strain of avian influenza, a highly pathogenic strain, was genetically closer to H6N2. Recent reports revealed that H6N2 is continuously evolving in different countries such as South Africa (Quan et al., 2019), Egypt (WHO, 2018a), India (Worldometer, 2020), and North America (Zanaty et al., 2019) due to genetic reassortment. It is gradually evolving from the low pathogenic form to the high pathogenic form and observed to overcome species barrier with interspecies genetic assortment (Kumar et al., 2018; Richard et al., 2002b; Gillim-Ross et al., 2008; FAO, 2019) and every possibility to evolve as a pandemic for human. Reports are available depicting human influenza virus arising due to genetic reassortment of avian influenza in China (Jiao et al., 2012; Ayora-Talavera, 2018; Karen, 2018; Li et al., 2020). These findings highlight the growing importance of the study in the current era, when the world is suffering from a pandemic.

Although molecular docking analysis is available for the identification of various drug molecules with avian influenza virus (Amir et al., 2011; Liu et al., 2015; Khan et al., 2017; Pal and Chakravarty, 2019b), this is the first report of molecular docking analysis with the immune response molecules responsible for antiviral immunity against avian influenza and is the basis for finding drug for a disease. A series of immune response molecules are responsible for providing antiviral immunity with their respective interaction in various pathways as we depicted through String and KEGG pathway analyses in our current study.

The future outcome for the current study is the possible utilization of the identified disease resistant genes (RIGI, TLR3, and TLR7) for the development of avian influenza–resistant chicken with the identified gene insert from duck through gene editing or a transgenic approach.

## Conclusion

RIG1 detects the virus that is present within the cytosol of infected cells (cell intrinsic recognition), whereas TLR3 detects virus-infected cells, and TLR7 detects viral RNA that has taken up into the endosomes of sentinel cells (cell-extrinsic recognition). TLR7 may be regarded as the promising gene for antiviral immunity with pronounced expression profiling in duck in contrast to other poultry birds. Molecular docking revealed RIGI, TLR3, and TLR7 as the promising genes conferring antiviral immunity against avian influenza. Point mutations have been detected in chicken TLR3 with respect to that of duck indicative of reduced antiviral immunity in chicken in comparison to duck utilization of the identified disease-resistant genes (RIGI, TLR3, and TLR7) for the development of avian influenza–resistant chicken with the identified gene insert from duck.

## Data Availability

The datasets presented in this study can be found in online repositories. The names of the repository/repositories and accession number(s) can be found in the article/Supplementary Material.
