# Supplementary material for: RIGI, TLR7, and TLR3 Genes Were Predicted to Have Immune Response Against Avian Influenza in Indigenous Ducks
Source: Front Mol Biosci. 2021 Dec 14;8:633283. doi: 10.3389/fmolb.2021.633283 (PMC8712727; doi:10.3389/fmolb.2021.633283)
Supplement: Supplementary file 1 [file Image1.pdf]

Supplementary Fig 1: PatchDock Analysis

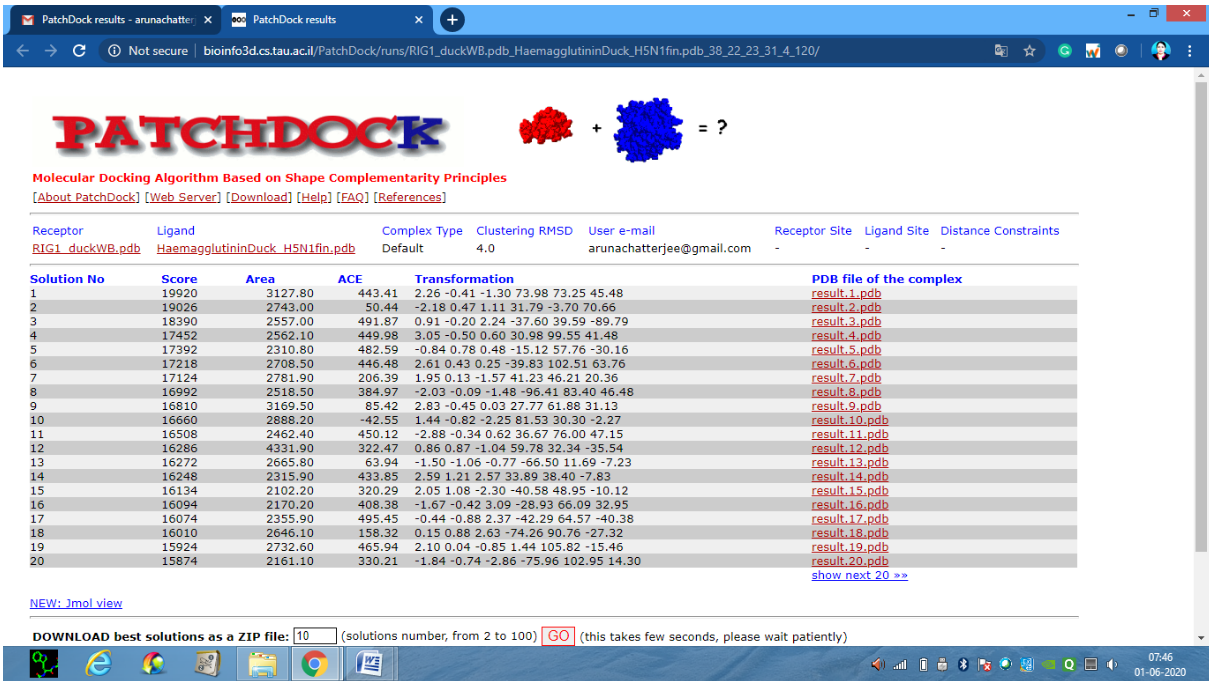

PatchDock results - arunachatter | PatchDock results

Not secure | bioinfo3d.cs.tau.ac.il/PatchDock/runs/RIG1_duckWB.pdb_HaemagglutininDuck_H5N1fin.pdb_38_22_23_31_4_120/

PATCHDOCK

Molecular Docking Algorithm Based on Shape Complementarity Principles

[About PatchDock] [Web Server] [Download] [Help] [FAQ] [References]

Receptor	Ligand	Complex Type	Clustering RMSD	User e-mail	Receptor Site	Ligand Site	Distance Constraints
RIG1_duckWB.pdb	HaemagglutininDuck_H5N1fin.pdb	Default	4.0	arunachatterjee@gmail.com	-	-	-

Solution No	Score	Area	ACE	Transformation	PDB file of the complex
1	19920	3127.80	443.41	2.26 -0.41 -1.30 73.98 73.25 45.48	result.1.pdb
2	19026	2743.00	50.44	-2.18 0.47 1.11 31.79 -3.70 70.66	result.2.pdb
3	18390	2557.00	491.87	0.91 -0.20 2.24 -37.60 39.59 -89.79	result.3.pdb
4	17452	2562.10	449.98	3.05 -0.50 0.60 30.98 99.55 41.48	result.4.pdb
5	17392	2310.80	482.59	-0.84 0.78 0.48 -15.12 57.76 -30.16	result.5.pdb
6	17218	2708.50	446.48	2.61 0.43 0.25 -39.83 102.51 63.76	result.6.pdb
7	17124	2781.90	206.39	1.95 0.13 -1.57 41.23 46.21 20.36	result.7.pdb
8	16992	2518.50	384.97	-2.03 -0.09 -1.48 -96.41 83.40 46.48	result.8.pdb
9	16810	3169.50	85.42	2.83 -0.45 0.03 27.77 61.88 31.13	result.9.pdb
10	16660	2888.20	-42.55	1.44 -0.82 -2.25 81.53 30.30 -2.27	result.10.pdb
11	16508	2462.40	450.12	-2.88 -0.34 0.62 36.67 76.00 47.15	result.11.pdb
12	16286	4331.90	322.47	0.86 0.87 -1.04 59.78 32.34 -35.54	result.12.pdb
13	16272	2665.80	63.94	-1.50 -1.06 -0.77 -66.50 11.69 -7.23	result.13.pdb
14	16248	2315.90	433.85	2.59 1.21 2.57 33.89 38.40 -7.83	result.14.pdb
15	16134	2102.20	320.29	2.05 1.08 -2.30 -40.58 48.95 -10.12	result.15.pdb
16	16094	2170.20	408.38	-1.67 -0.42 3.09 -28.93 66.09 32.95	result.16.pdb
17	16074	2355.90	495.45	-0.44 -0.88 2.37 -42.29 64.57 -40.38	result.17.pdb
18	16010	2646.10	158.32	0.15 0.88 2.63 -74.26 90.76 -27.32	result.18.pdb
19	15924	2732.60	465.94	2.10 0.04 -0.85 1.44 105.82 -15.46	result.19.pdb
20	15874	2161.10	330.21	-1.84 -0.74 -2.86 -75.96 102.95 14.30	result.20.pdb

show next 20 »»

NEW: Jmol view

DOWNLOAD best solutions as a ZIP file: 10 (solutions number, from 2 to 100) GO (this takes few seconds, please wait patiently)

07:46 01-06-2020

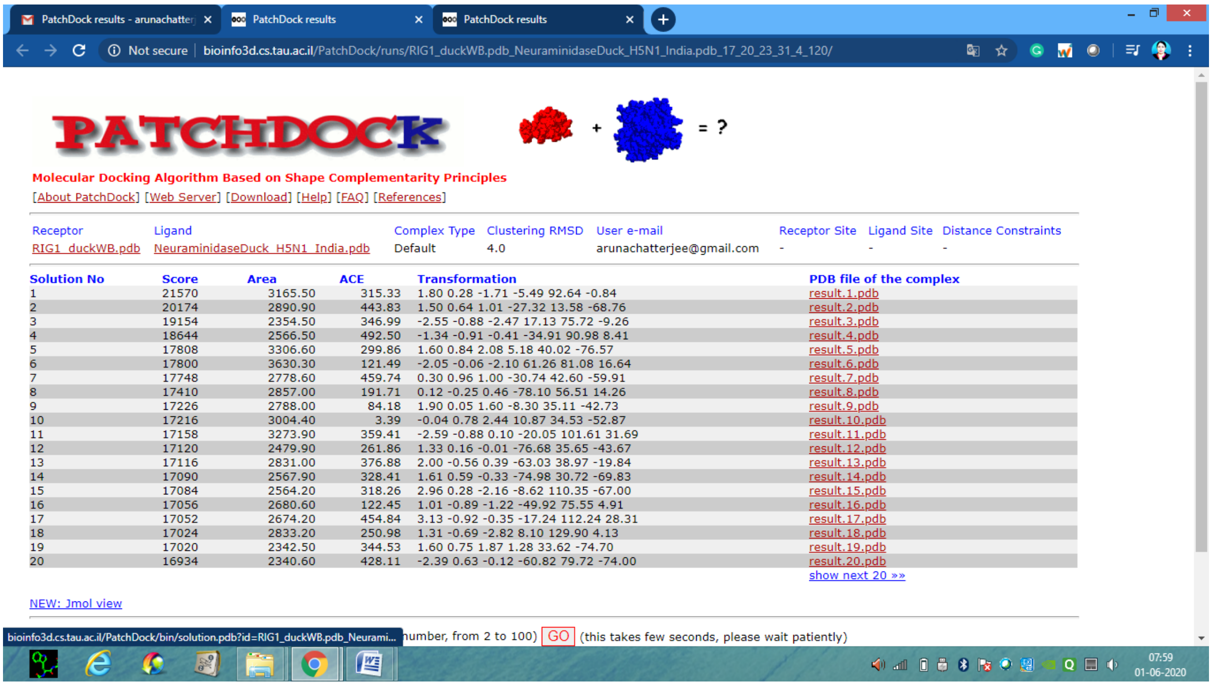

PatchDock results - arunachatter | PatchDock results | PatchDock results

Not secure | bioinfo3d.cs.tau.ac.il/PatchDock/runs/RIG1_duckWB.pdb_NeuraminidaseDuck_H5N1_India.pdb_17_20_23_31_4_120/

PATCHDOCK

Molecular Docking Algorithm Based on Shape Complementarity Principles

[About PatchDock] [Web Server] [Download] [Help] [FAQ] [References]

Receptor	Ligand	Complex Type	Clustering RMSD	User e-mail	Receptor Site	Ligand Site	Distance Constraints
RIG1_duckWB.pdb	NeuraminidaseDuck_H5N1_India.pdb	Default	4.0	arunachatterjee@gmail.com	-	-	-

Solution No	Score	Area	ACE	Transformation	PDB file of the complex
1	21570	3165.50	315.33	1.80 0.28 -1.71 -5.49 92.64 -0.84	result.1.pdb
2	20174	2890.90	443.83	1.50 0.64 1.01 -27.32 13.58 -68.76	result.2.pdb
3	19154	2354.50	346.99	-2.55 -0.88 -2.47 17.13 75.72 -9.26	result.3.pdb
4	18644	2566.50	492.50	-1.34 -0.91 -0.41 -34.91 90.98 8.41	result.4.pdb
5	17808	3306.60	299.86	1.60 0.84 2.08 5.18 40.02 -76.57	result.5.pdb
6	17800	3630.30	121.49	-2.05 -0.06 -2.10 61.26 81.08 16.64	result.6.pdb
7	17748	2778.60	459.74	0.30 0.96 1.00 -30.74 42.60 -59.91	result.7.pdb
8	17410	2857.00	191.71	0.12 -0.25 0.46 -78.10 56.51 14.26	result.8.pdb
9	17226	2788.00	84.18	1.90 0.05 1.60 -8.30 35.11 -42.73	result.9.pdb
10	17216	3004.40	3.39	-0.04 0.78 2.44 10.87 34.53 -52.87	result.10.pdb
11	17158	3273.90	359.41	-2.59 -0.88 0.10 -20.05 101.61 31.69	result.11.pdb
12	17120	2479.90	261.86	1.33 0.16 -0.01 -76.68 35.65 -43.67	result.12.pdb
13	17116	2831.00	376.88	2.00 -0.56 0.39 -63.03 38.97 -19.84	result.13.pdb
14	17090	2567.90	328.41	1.61 0.59 -0.33 -74.98 30.72 -69.83	result.14.pdb
15	17084	2564.20	318.26	2.96 0.28 -2.16 -8.62 110.35 -67.00	result.15.pdb
16	17056	2680.60	122.45	1.01 -0.89 -1.22 -49.92 75.55 4.91	result.16.pdb
17	17052	2674.20	454.84	3.13 -0.92 -0.35 -17.24 112.24 28.31	result.17.pdb
18	17024	2833.20	250.98	1.31 -0.69 -2.82 8.10 129.90 4.13	result.18.pdb
19	17020	2342.50	344.53	1.60 0.75 1.87 1.28 33.62 -74.70	result.19.pdb
20	16934	2340.60	428.11	-2.39 0.63 -0.12 -60.82 79.72 -74.00	result.20.pdb

show next 20 »»

NEW: Jmol view

bioinfo3d.cs.tau.ac.il/PatchDock/bin/solution.pdb?id=RIG1_duckWB.pdb_Neurami... umber, from 2 to 100) GO (this takes few seconds, please wait patiently)

07:59 01-06-2020

PatchDock results - arunachatter | PatchDock results

Not secure | bioinfo3d.cs.tau.ac.il/PatchDock/runs/TLR7_duckWB.pdb_HaemagglutininDuck_H5N1fin.pdb_35_25_23_31_4_120/

PATCHDOCK

+ = ?

Molecular Docking Algorithm Based on Shape Complementarity Principles

[About PatchDock] [Web Server] [Download] [Help] [FAQ] [References]

Receptor	Ligand	Complex Type	Clustering RMSD	User e-mail	Receptor Site	Ligand Site	Distance Constraints
TLR7_duckWB.pdb	HaemagglutininDuck_H5N1fin.pdb	Default	4.0	arunachatterjee@gmail.com	-	-	-

Solution No	Score	Area	ACE	Transformation	PDB file of the complex
1	20532	3179.20	427.90	-2.85 1.38 0.94 61.56 47.54 102.98	result.1.pdb
2	19446	4075.50	209.79	-2.48 -0.22 -3.07 57.68 136.83 148.04	result.2.pdb
3	19438	3424.80	-329.49	-3.03 -0.85 -2.66 38.76 70.67 117.11	result.3.pdb
4	19338	2974.00	-122.87	-2.25 -0.27 -3.04 54.25 150.25 138.56	result.4.pdb
5	18556	2417.00	242.45	-3.01 -0.80 0.63 143.12 172.12 100.83	result.5.pdb
6	18414	2739.60	290.01	-1.57 -0.16 -1.95 116.76 133.87 58.60	result.6.pdb
7	18380	2947.50	298.25	2.18 -0.92 0.90 64.54 202.61 95.78	result.7.pdb
8	18320	3113.10	349.95	-2.04 -0.41 0.38 138.46 63.06 146.19	result.8.pdb
9	18230	3206.60	265.67	2.76 -0.95 -0.19 115.67 113.52 138.01	result.9.pdb
10	18010	3075.00	453.65	1.30 0.29 1.00 85.76 174.31 97.04	result.10.pdb
11	17662	3272.40	396.55	1.16 0.69 -2.33 80.53 92.39 56.41	result.11.pdb
12	17514	3492.60	375.71	1.68 0.63 -1.85 157.30 93.95 114.59	result.12.pdb
13	17488	3129.30	300.71	1.98 0.84 -1.95 162.79 104.73 132.68	result.13.pdb
14	17470	2954.80	342.35	-1.38 0.12 1.98 198.00 167.17 86.11	result.14.pdb
15	17448	2800.00	216.10	1.36 0.86 -1.60 177.25 102.07 110.04	result.15.pdb
16	17442	2865.10	334.54	1.48 0.43 1.15 75.49 171.42 106.37	result.16.pdb
17	17326	2976.70	-5.12	1.46 1.38 0.47 64.01 120.33 64.71	result.17.pdb
18	17308	2511.10	150.48	0.62 -1.19 -1.22 114.08 194.85 35.33	result.18.pdb
19	17290	2443.90	302.58	2.17 -0.59 -0.94 120.62 135.43 145.43	result.19.pdb
20	17250	2651.00	140.46	2.98 0.77 -3.12 136.40 106.99 164.17	result.20.pdb

show next 20 »»

NEW: Jmol view

DOWNLOAD best solutions as a ZIP file: 10 (solutions number, from 2 to 100) GO (this takes few seconds, please wait patiently)

08:03
01-06-2020

PatchDock results - arunachatter | PatchDock results

Not secure | bioinfo3d.cs.tau.ac.il/PatchDock/runs/TLR7_duckWB.pdb_NeuraminidaseDuck_H5N1_Indiafin.pdb_13_25_23_31_4_120/

PATCHDOCK

+ = ?

Molecular Docking Algorithm Based on Shape Complementarity Principles

[About PatchDock] [Web Server] [Download] [Help] [FAQ] [References]

Receptor	Ligand	Complex Type	Clustering RMSD	User e-mail	Receptor Site	Ligand Site	Distance Constraints
TLR7_duckWB.pdb	NeuraminidaseDuck_H5N1_Indiafin.pdb	Default	4.0	arunachatterjee@gmail.com	-	-	-

Solution No	Score	Area	ACE	Transformation	PDB file of the complex
1	20600	2724.60	442.80	-2.79 -0.80 -1.64 124.87 141.90 124.60	result.1.pdb
2	20014	3028.50	374.32	-1.71 0.34 -2.86 161.57 74.53 68.79	result.2.pdb
3	19974	3106.50	413.86	1.27 -0.40 2.51 161.84 88.73 120.45	result.3.pdb
4	19482	2545.90	315.47	2.91 1.07 2.34 109.48 93.34 46.71	result.4.pdb
5	19050	3874.80	483.06	-2.63 1.30 1.46 92.60 104.33 49.47	result.5.pdb
6	18832	2825.80	399.16	0.39 1.10 -2.54 134.05 129.01 69.44	result.6.pdb
7	18548	3937.00	392.49	2.10 -1.25 -2.79 132.97 123.74 138.26	result.7.pdb
8	18516	2646.20	281.34	-0.01 -1.38 2.92 108.91 107.13 159.95	result.8.pdb
9	18318	2657.70	158.18	-1.78 -0.63 -1.73 168.55 138.20 143.88	result.9.pdb
10	18208	2440.90	393.72	-0.95 -0.14 -2.16 149.40 122.66 81.46	result.10.pdb
11	18112	2587.80	344.56	-0.06 -0.28 1.12 103.07 121.71 114.52	result.11.pdb
12	18022	2738.50	-237.20	-1.76 0.25 1.05 82.86 94.07 35.58	result.12.pdb
13	17970	3179.50	410.85	-2.14 0.92 2.11 108.06 66.39 61.96	result.13.pdb
14	17910	3001.70	374.16	-2.37 1.19 1.04 87.90 101.52 52.63	result.14.pdb
15	17852	2806.30	355.03	-1.50 -0.07 -0.28 80.31 194.99 96.75	result.15.pdb
16	17800	4054.70	445.62	3.03 -1.04 -1.94 127.61 139.38 129.81	result.16.pdb
17	17798	2839.50	297.64	-1.56 -1.14 0.38 91.20 176.97 139.24	result.17.pdb
18	17796	2578.10	451.37	2.50 0.08 -2.33 134.91 133.70 76.04	result.18.pdb
19	17774	3116.60	172.89	1.35 0.91 -2.75 125.54 120.86 44.60	result.19.pdb
20	17696	2770.90	-241.10	1.54 0.88 1.76 161.51 102.34 7.13	result.20.pdb

show next 20 »»

NEW: Jmol view

DOWNLOAD best solutions as a ZIP file: 10 (solutions number, from 2 to 100) GO (this takes few seconds, please wait patiently)

08:12
01-06-2020

PatchDock results - arunachatter | PatchDock results

Not secure | bioinfo3d.cs.tau.ac.il/PatchDock/runs/Tlr3wb__.pdb_NeuraminidaseDuck_H5N1_Indiafin.pdb_20_26_23_31_4_120/

PATCHDOCK

Molecular Docking Algorithm Based on Shape Complementarity Principles

[About PatchDock] [Web Server] [Download] [Help] [FAQ] [References]

Receptor	Ligand	Complex Type	Clustering RMSD	User e-mail	Receptor Site	Ligand Site	Distance Constraints
Tlr3wb__.pdb	NeuraminidaseDuck_H5N1_Indiafin.pdb	Default	4.0	arunachatterjee@gmail.com	-	-	-

Solution No	Score	Area	ACE	Transformation	PDB file of the complex
1	21120	3243.20	-72.95	0.16 0.57 0.85 75.16 36.49 71.45	result.1.pdb
2	20800	3620.90	209.67	-1.94 -0.24 0.05 58.91 108.44 86.11	result.2.pdb
3	20294	3193.50	110.25	-1.42 -1.33 0.67 91.98 103.12 126.06	result.3.pdb
4	19488	2909.80	253.93	-1.83 -0.69 -1.31 125.06 137.80 142.60	result.4.pdb
5	19474	2980.00	320.17	2.49 -0.57 -1.94 122.97 148.68 109.43	result.5.pdb
6	19432	2760.30	212.17	-2.29 0.39 -0.26 76.09 119.46 42.34	result.6.pdb
7	19044	2558.70	294.02	-2.03 0.53 -2.25 98.52 140.23 52.56	result.7.pdb
8	18578	4009.10	-516.61	-0.31 -1.01 -1.88 67.49 121.87 115.45	result.8.pdb
9	18276	3155.80	-198.35	-2.38 -0.75 -0.23 72.39 117.88 98.27	result.9.pdb
10	18196	2355.10	270.46	0.63 1.01 -1.86 101.76 131.78 63.56	result.10.pdb
11	18176	2476.70	265.90	-2.58 -0.27 0.46 82.81 96.83 118.80	result.11.pdb
12	18142	2898.90	264.65	-2.77 -0.26 -0.83 100.07 146.13 108.12	result.12.pdb
13	18124	2828.30	404.45	-1.67 -0.32 0.39 61.47 90.62 92.63	result.13.pdb
14	18096	2706.90	177.01	-1.84 -0.47 -1.45 127.89 141.26 137.30	result.14.pdb
15	17968	2771.20	234.62	-0.08 0.14 -0.88 72.32 124.95 100.76	result.15.pdb
16	17830	2561.20	164.69	-2.69 -1.28 -0.56 89.73 115.71 123.11	result.16.pdb
17	17820	2486.90	48.92	-0.85 -1.04 -1.09 130.33 113.65 165.44	result.17.pdb
18	17732	2212.50	212.10	-2.91 0.57 1.43 90.08 60.92 57.81	result.18.pdb
19	17714	3385.20	-342.55	-2.03 -1.36 -1.42 81.71 118.75 110.82	result.19.pdb
20	17490	3545.60	-175.31	-0.92 -0.54 -0.34 82.82 118.62 144.13	result.20.pdb

show next 20 »»

NEW: Jmol view

DOWNLOAD best solutions as a ZIP file: 10 (solutions number, from 2 to 100) GO (this takes few seconds, please wait patiently)

08:26 01-06-2020

PatchDock results - arunachatter | PatchDock results

Not secure | bioinfo3d.cs.tau.ac.il/PatchDock/runs/Tlr3wb__.pdb_HaemagglutininDuck_H5N1fin.pdb_51_26_23_31_4_120/

PATCHDOCK

Molecular Docking Algorithm Based on Shape Complementarity Principles

[About PatchDock] [Web Server] [Download] [Help] [FAQ] [References]

Receptor	Ligand	Complex Type	Clustering RMSD	User e-mail	Receptor Site	Ligand Site	Distance Constraints
Tlr3wb__.pdb	HaemagglutininDuck_H5N1fin.pdb	Default	4.0	arunachatterjee@gmail.com	-	-	-

Solution No	Score	Area	ACE	Transformation	PDB file of the complex
1	22880	4135.70	342.57	-2.37 1.21 0.26 52.42 58.95 115.16	result.1.pdb
2	21152	5091.40	215.62	1.00 -0.99 1.82 89.07 90.50 77.84	result.2.pdb
3	20252	3444.50	374.51	-2.28 0.44 0.09 35.97 84.37 114.19	result.3.pdb
4	20022	3685.80	382.51	2.98 0.77 2.43 124.06 47.05 140.19	result.4.pdb
5	19116	2603.60	478.92	-2.24 0.29 -0.72 107.22 89.44 136.09	result.5.pdb
6	18996	2794.90	413.25	-2.71 0.41 2.89 103.11 130.13 167.17	result.6.pdb
7	18934	3650.10	264.24	3.12 1.29 1.12 56.18 53.90 110.91	result.7.pdb
8	18656	3797.60	438.78	-0.44 0.97 2.36 121.30 167.39 58.27	result.8.pdb
9	18580	2349.90	378.94	0.52 0.14 0.45 106.35 161.44 8.34	result.9.pdb
10	18436	2380.60	470.53	-1.06 1.18 -1.48 120.32 148.24 121.45	result.10.pdb
11	18404	3336.00	337.38	3.10 1.00 2.48 141.87 44.23 130.90	result.11.pdb
12	18110	3192.90	335.60	-2.54 1.22 0.50 58.69 54.72 118.70	result.12.pdb
13	18104	2441.10	368.25	2.92 0.44 -0.59 126.41 163.21 171.81	result.13.pdb
14	18020	2270.80	-38.45	1.63 -0.29 1.15 76.23 169.94 100.49	result.14.pdb
15	17986	2598.90	211.86	-0.25 -1.08 -2.86 153.62 152.54 47.04	result.15.pdb
16	17896	4186.60	138.24	-0.56 -1.03 0.09 73.53 43.59 81.28	result.16.pdb
17	17778	3236.90	303.80	1.66 0.94 1.37 51.53 153.10 122.74	result.17.pdb
18	17722	2350.90	359.11	-0.69 0.05 2.40 126.17 179.04 99.05	result.18.pdb
19	17714	3300.60	415.72	-0.62 0.43 2.03 115.10 165.04 76.64	result.19.pdb
20	17672	2596.50	81.35	-1.62 1.03 1.85 181.24 145.07 134.27	result.20.pdb

show next 20 »»

NEW: Jmol view

DOWNLOAD best solutions as a ZIP file: 10 (solutions number, from 2 to 100) GO (this takes few seconds, please wait patiently)

08:29 01-06-2020

PatchDock results - arunachatter | PatchDock results

Not secure | bioinfo3d.cs.tau.ac.il/PatchDock/runs/modeller_RIG_I_4A2W.pdb_HaemagglutininDuck_H5N1fin.pdb_28_15_14_3_5_120/

PATCHDOCK

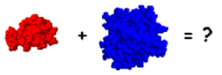

Molecular Docking Algorithm Based on Shape Complementarity Principles

[About PatchDock] [Web Server] [Download] [Help] [FAQ] [References]

Receptor	Ligand	Complex Type	Clustering RMSD	User e-mail	Receptor Site	Ligand Site	Distance Constraints
modeller_RIG_I_4A2W.pdb	HaemagglutininDuck_H5N1fin.pdb	Default	4.0	arunachatterjee@gmail.com	-	-	-

Solution No	Score	Area	ACE	Transformation	PDB file of the complex
1	19366	4211.80	141.50	1.13 0.71 -0.48 69.89 -33.98 -52.97	result.1.pdb
2	19102	2741.30	435.97	-0.31 0.28 0.21 -27.80 -88.78 -62.19	result.2.pdb
3	18180	2864.50	464.32	1.30 0.04 -1.13 -21.22 -60.15 -28.32	result.3.pdb
4	18166	3147.60	254.53	-1.49 -0.05 2.98 20.36 7.25 -38.97	result.4.pdb
5	18162	2754.00	194.09	-0.48 -0.39 -0.62 -17.20 -75.84 -81.02	result.5.pdb
6	17982	2976.30	353.91	-1.09 -0.29 -0.37 -31.74 -67.88 -42.55	result.6.pdb
7	17458	3152.30	346.14	-2.08 0.30 -2.13 2.13 -67.63 22.70	result.7.pdb
8	17076	2869.60	226.32	0.07 -0.10 -2.35 2.74 -84.69 -89.02	result.8.pdb
9	16890	2392.90	323.29	-2.85 -0.44 0.23 49.92 -49.16 22.07	result.9.pdb
10	16842	2949.00	495.07	-2.28 -0.21 -2.68 -52.15 -63.24 -17.71	result.10.pdb
11	16816	3306.50	287.26	2.36 0.81 -0.35 -34.15 23.12 -24.71	result.11.pdb
12	16688	3376.30	346.44	-2.27 -0.29 -2.19 -66.97 -18.02 36.00	result.12.pdb
13	16658	4048.40	177.98	2.51 -0.53 -0.99 81.23 -40.73 38.93	result.13.pdb
14	16556	2789.90	262.70	-3.14 0.15 0.68 -17.07 -44.37 -27.46	result.14.pdb
15	16550	2651.90	419.13	-1.87 0.69 -2.15 -47.34 -42.39 -32.33	result.15.pdb
16	16534	2160.60	279.39	-1.24 -0.34 -1.20 -85.14 -53.48 -39.74	result.16.pdb
17	16526	2272.80	485.92	-2.17 0.39 -0.01 -70.24 -114.23 29.77	result.17.pdb
18	16508	3068.10	483.45	-2.32 0.94 1.18 30.19 -76.81 21.96	result.18.pdb
19	16492	2567.50	496.12	2.64 -0.39 1.09 -72.57 -0.93 9.98	result.19.pdb
20	16474	2819.10	446.41	2.92 -0.77 0.65 40.82 2.07 12.92	result.20.pdb

show next 20 »»

NEW: Jmol view

DOWNLOAD best solutions as a ZIP file: 10 (solutions number, from 2 to 100) GO (this takes few seconds, please wait patiently)

06:20 04-06-2020

PatchDock results - arunachatter | PatchDock results

Not secure | bioinfo3d.cs.tau.ac.il/PatchDock/runs/modeller_RIG_I_4A2W.pdb_NeuraminidaseDuck_H5N1_Indiafin.pdb_6_54_3_4_5_120/

PATCHDOCK

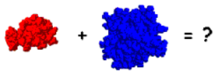

Molecular Docking Algorithm Based on Shape Complementarity Principles

[About PatchDock] [Web Server] [Download] [Help] [FAQ] [References]

Receptor	Ligand	Complex Type	Clustering RMSD	User e-mail	Receptor Site	Ligand Site	Distance Constraints
modeller_RIG_I_4A2W.pdb	NeuraminidaseDuck_H5N1_Indiafin.pdb	Default	4.0	arunachatterjee@gmail.com	-	-	-

Solution No	Score	Area	ACE	Transformation	PDB file of the complex
1	20862	3070.40	256.89	-2.29 -0.55 -0.62 0.43 -23.73 21.32	result.1.pdb
2	20340	3020.60	-73.24	-2.25 -0.87 -1.68 -10.60 -35.42 -16.68	result.2.pdb
3	19620	3340.50	193.25	-1.55 0.14 2.17 35.47 -120.13 2.60	result.3.pdb
4	19478	2947.00	-208.00	2.97 -0.40 -2.42 68.22 -35.66 1.71	result.4.pdb
5	19366	3186.70	201.51	-3.09 -0.98 2.97 13.29 -53.74 -15.65	result.5.pdb
6	19358	3410.70	285.33	2.21 0.16 -0.75 -15.46 -46.93 -21.25	result.6.pdb
7	19232	2806.70	99.59	-0.86 -0.26 0.93 -52.98 -52.65 -15.52	result.7.pdb
8	18744	2943.90	252.31	-1.83 -1.24 -0.90 -34.35 -34.59 29.37	result.8.pdb
9	18662	3585.20	264.15	2.17 -0.41 -1.68 -29.81 -42.81 7.03	result.9.pdb
10	18590	3524.30	-23.53	-0.60 -0.17 -0.33 -40.55 -39.40 -35.78	result.10.pdb
11	18466	2972.10	239.46	3.09 -0.91 1.86 -19.15 -79.92 -18.60	result.11.pdb
12	18370	3248.80	66.08	1.43 -0.06 2.70 30.19 -95.98 3.28	result.12.pdb
13	18314	2674.40	-209.82	0.81 -0.11 -2.76 1.42 -9.36 -23.35	result.13.pdb
14	18246	2997.30	-12.47	1.88 -0.54 2.62 39.50 -34.26 -32.07	result.14.pdb
15	18056	3373.70	232.26	-2.62 0.89 1.47 7.24 -83.43 -42.31	result.15.pdb
16	17990	2702.40	355.41	-2.04 -0.23 1.69 12.26 -112.50 10.47	result.16.pdb
17	17960	2926.80	-314.09	2.66 0.06 -0.97 -5.73 -36.80 -17.02	result.17.pdb
18	17872	3568.20	-365.38	-1.32 0.70 -2.80 15.68 -40.56 -61.81	result.18.pdb
19	17848	3308.60	292.43	-0.53 -0.00 2.08 -9.30 -78.59 -18.73	result.19.pdb
20	17838	2222.00	343.04	1.97 -0.27 3.00 91.51 -51.60 -55.65	result.20.pdb

show next 20 »»

NEW: Jmol view

DOWNLOAD best solutions as a ZIP file: 10 (solutions number, from 2 to 100) GO (this takes few seconds, please wait patiently)

15:28 04-06-2020
